# The Secretive Liaison of Particulate Matter and SARS-CoV-2. A Hypothesis and Theory Investigation

**DOI:** 10.3389/fgene.2020.579964

**Published:** 2020-11-09

**Authors:** Ada Mescoli, Giangabriele Maffei, Gelsomina Pillo, Giuseppe Bortone, Stefano Marchesi, Elena Morandi, Andrea Ranzi, Francesca Rotondo, Stefania Serra, Monica Vaccari, Stefano Zauli Sajani, Maria Grazia Mascolo, Miriam Naomi Jacobs, Annamaria Colacci

**Affiliations:** ^1^Department of Experimental, Diagnostic and Specialty Medicine, Section of Cancerology, University of Bologna, Bologna, Italy; ^2^Agency for Prevention, Environment and Energy (Arpae), Emilia-Romagna, Italy; ^3^Department of Toxicology, Centre for Radiation, Chemical and Environmental Hazards Public Health England, Chilton, United Kingdom

**Keywords:** COVID-19, particulate matter, SARS-CoV-2, environmental pollution, cytokine storm, molecular signatures, receptors cross-talk, molecular mechanisms

## Abstract

As the novel coronavirus disease sweeps across the world, there is growing speculation on the role that atmospheric factors may have played on the different distribution of SARS-CoV-2, and on the epidemiological characteristics of COVID-19. Knowing the role that environmental factors play in influenza virus outbreaks, environmental pollution and, in particular, atmospheric airborne (particulate matter, PM) has been considered as a potential key factor in the spread and mortality of COVID-19. A possible role of the PM as the virus carrier has also been debated. The role of PM in exacerbating respiratory and cardiovascular disease has been well recognized. Accumulating evidence support the hypothesis that PM can trigger inflammatory response at molecular, cellular and organ levels. On this basis, we developed the hypothesis that PM may play a role as a booster of COVID-19 rather than as a carrier of SARS-CoV-2. To support our hypothesis, we analyzed the molecular signatures detected in cells exposed to PM samples collected in one of the most affected areas by the COVID-19 outbreak, in Italy. T47D human breast adenocarcinoma cells were chosen to explore the global gene expression changes induced by the treatment with organic extracts of PM 2.5. The analysis of the KEGG’s pathways showed modulation of several gene networks related to the leucocyte transendothelial migration, cytoskeleton and adhesion system. Three major biological process were identified, including coagulation, growth control and immune response. The analysis of the modulated genes gave evidence for the involvement of PM in the endothelial disease, coagulation disorders, diabetes and reproductive toxicity, supporting the hypothesis that PM, directly or through molecular interplay, affects the same molecular targets as so far known for SARS-COV-2, contributing to the cytokines storm and to the aggravation of the symptoms triggered by COVID-19. We provide evidence for a plausible cooperation of receptors and transmembrane proteins, targeted by PM and involved in COVID-19, together with new insights into the molecular interplay of chemicals and pathogens that could be of importance for sustaining public health policies and developing new therapeutic approaches.

## Introduction

The rapid and dramatic spread of the novel coronavirus disease 2019 (COVID-19), not surprisingly, continues to generate great interest and attract attention among researchers and scientists of all disciplines, with the aim of better understanding the way the virus spreads, considering its mechanisms of interaction with the host, and investigating all possible factors, which can affect the spread and severity of the disease.

Knowing the role that environmental factors play in influenza virus outbreaks, several speculations have been made regarding the role that atmospheric factors may have played on the distribution of SARS-CoV-2 and on the epidemiological characteristics of COVID-19 (Baldini et al., 2020; Conticini et al., 2020; Contini and Costabile, 2020; Sajadi et al., 2020).

The first input came from the analysis of meteorological variables, which indicated the most significant spread of the infection east-west along a geographical stretch (corridor) within 30–50 N′, characterized by similar weather conditions, with low temperature, mild diurnal temperature range and low relative humidity (Sajadi et al., 2020).

This geographical stretch includes regions and areas characterized by high levels of air pollution. Therefore, it was speculated that environmental pollution had played a key role in the spread of the COVID-19 disease, and even debated whether the atmospheric airborne (PM) may act as a carrier of the virus SARS-CoV-2 (Contini and Costabile, 2020; Conticini et al., 2020; Miyashita et al., 2020). Using Italian data (until 21st March 2020) and comparing lethality in Lombardy and Emilia-Romagna with the rest of Italy, Conticini et al. (2020) have speculated that chronic exposure to air pollution can increase the susceptibility for respiratory disease, and might render an individual more susceptible to SARS-CoV-2 and other respiratory infections, when compared to residents in areas with lower pollution.

However, it should be noted that the evidence for causality described in Conticini et al. (2020), does not take into account the effect of the lockdown on the dramatic reduction in contagion.

Generally speaking, biological agents can travel adsorbed or bound to solid or liquid particles suspended in the air. The actual possibility of maintaining the infectious potential strictly depends on the specific characteristics of the pathogen and particle considered.

In particular, the transmission of SARS-CoV-2 in humans occurs mainly through the droplets exhaled by an infected person in the immediate vicinity (<1–2 m) and/or, in a mediated way, through contact with contaminated surfaces and fomites (World Health Organization [WHO], 2020).

While the role of larger droplets (respiratory droplet, conventionally defined as those of size > 5 μm) in the contagion is well recognized, more uncertain is the role of micro-droplets (droplet nuclei – henceforth called bioaerosol), which several studies have shown to be produced in considerable numbers both in association with coughing and sneezing and in conditions of normal breathing or slight breathlessness. The dimensions of this bioaerosol can be extremely small (up to the size of the virus - between 100 and 200 nm) and for this reason can remain in suspension in the atmosphere for a long time (Bourouiba, 2020). These micro-droplets could, therefore, in theory be inhaled by people not in the immediate vicinity of infected persons and can interact with fine particles, solid or liquid, always present both in outdoor and indoor environments. While the transmission through the bioaerosol is considered of importance in indoor environments, it is believed that when viruses are expelled as bioaerosol or droplets from the respiratory tract in outdoor environments they tend to be inactivated due to rapid evaporation of water content and incoming solar radiation (Tseng and Li, 2005; Parienta et al., 2011).

It has been emphasized, however, that there is a need to explore the role of the physico-chemical properties of aerosol particles in inducing inflammation and oxidative stress as well as the toxicological activity associated with the chemical composition of airborne particles (Contini and Costabile, 2020).

PM can be considered as a paradigmatic example of an environmental mixture composed of airborne solid particles derived from both natural and anthropogenic sources. The concentration of PM is often used as an indicator of pollution level and represents a key factor for the assessments of the health risk from air pollution.

The respiratory system is the initial site of PM deposition and, as a consequence, the first target of PM health effects. The PM exposure can induce airway inflammation (Ghio et al., 2012; Darrow et al., 2014) and can affect the lung development (Gauderman et al., 2015), impairing the lung function in both children and adults. In addition, the PM exposure is related to the onset and exacerbation of obstructive lung diseases, including chronic obstructive pulmonary disease (COPD) and asthma, which represent important causes of morbidity and mortality worldwide (Peel et al., 2005; Cai et al., 2014; Jacquemin et al., 2015). Besides chronic asthma and pulmonary insufficiency, the long-term effects associated with air pollution include cardiovascular diseases (COMEAP, 2018), and cardiovascular mortality (Manisalidis et al., 2020). Positive associations between PM exposures and lung cancer incidence and mortality have been found, leading to the classification of PM as human carcinogen (Hamra et al., 2014). There is accumulating evidence that inflammatory mechanisms underpin PM exposure-related adverse outcomes (Wu et al., 2018).

We have previously described the effects of PM and its components in several *in vitro* cell models, elucidating key events at the molecular and cellular levels related to adverse outcomes, including molecular signatures sustaining the development of vasculopathy and vasculitis in auto-immune disease, or related to the idiopathic pulmonary fibrosis (Colacci et al., 2014; Vaccari et al., 2015). We have also found that the toxicological behavior of PM was strictly related to the content of PM components, such as the polycyclic aromatic hydrocarbons (PAHs). Indeed, PM can induce cell malignant transformation *in vitro* only when the concentration of PAHs exceeds the cells ability to implement its adaptive or detoxicant response (Vaccari et al., 2015; Mascolo et al., 2018; Serra et al., 2019).

On the basis of our results, we speculated that the binding of PM components, such as PAHs, to the Aryl hydrocarbon Receptor (AhR) triggers an immune-mediated response, leading to the activation of molecular pathways sustaining inflammation, through the release of several cytokines as signal mediators (Mascolo et al., 2018). Cytokines play a key role in the innate immune response against pathogens or toxic substances and have a complex regulatory effect on the inflammation and immune system.

The overproduction of early response cytokines, such as tumor necrosis factor (TNF), interleukin-6 (IL-6) and interleukin-1β (IL-1β), has been described in patients severely affected by COVID-19. This cytokine storm is associated with an increased risk of vascular permeability, multiorgan failure and death (Tisoncik et al., 2012).

SARS-CoV-2 induces inflammation by disrupting the renin-angiotensin axis (RAS), through the binding to the angiotensin-converting enzyme 2 receptor (ACE2), which negatively regulates RAS. ACE2 is widely distributed in human organs. In lung, it is highly expressed in type II alveolar cells, which play a key role in innate immunity and in repairing lung after injury. ACE2 represents the functional receptor for both SARS-CoV and SARS-CoV-2 and their gateway to the host cell. In animal models, the infection with SARS-CoV, but also with the avian influenza A H5N1 virus, leads to the increase of lung angiotensin II levels and to a decrease of the ACE2 protein levels, showing the downregulation of ACE2 following the infection with respiratory viruses.

The increase of cytokines involved in systemic inflammation, following the exposure to PM, has been related to the increase of the risk and severity for cardiovascular disease in humans (Pope et al., 2016; Miyashita et al., 2020), and it has been confirmed in animal models, where a PM-related mechanism involving the renin-angiotensin-bradykinin system has been postulated (Aztatzi-Aguilar et al., 2015; Lin et al., 2018; Sharma et al., 2019).

With the view of the mechanism of action of SARS-CoV-2 leading to severe pulmonary effects, such as interstitial pneumonia, cardiovascular effects and, as described more recently, autoimmune vascular diseases, we developed the hypothesis that PM plays a role as a booster of COVID-19 rather than as a carrier of SARS-CoV2, interplaying at the molecular level, amplifying the immune-mediated response and contributing to the abnormal production of immune system mediators in several COVID+ patients.

To support our hypothesis, we reviewed data from literature reports, including our previous published data, and analyzed the molecular signatures detected in cells exposed to PM samples collected in one of the most affected areas by the COVID-19 outbreak, in Italy. The results from the analysis of these original data are presented and discussed in this paper.

PM samples were collected in periods prior to the COVID-19 outbreak, at several sites in the surroundings of Bologna (Emilia-Romagna, Italy), which is located in the Po valley. The Po valley in northern Italy is characterized by a high density of anthropogenic emissions- and it is considered one of the most polluted areas in Europe due to particular climate conditions and geographical location.

The average annual concentration in the area has been calculated to be 21–23 μg/m^3^ PM2.5, ranging 14–28 μg/m^3^. However, particular meteorological conditions, characterized by atmospheric stagnation in winter can lead to peaks of air pollution, persisting for several days and largely exceeding the limits set by the European Unit (EU) regulation for air quality. The same area in summer is affected by high levels of ozone. Therefore, samples were collected in both winter and summer, to consider the seasonal influence on health outcomes. The effects of PM were analyzed in a T47D cell line model by using an integrated approach, coupling the *in vitro* model with toxicogenomics. T47D human breast adenocarcinoma cells were chosen to explore the global gene expression changes induced by the treatment with organic extracts of PM2.5, which represents the fraction that more easily enters the respiratory tract. T47D cells express a functional AhR, plus several other hormone receptors, including estrogen receptors, ERalpha, ERbeta, the androgen receptor (AR), progesterone receptor (PR), glucocorticoid receptor, prolactin receptor and the growth hormone receptor. T47D cells also express both angiotensin type 1 receptors, AT1 and AT2 (Gui et al., 1997; Inwang et al., 1997). Recently, T47D cells have been reported to express high levels of mitochondrial assembly receptor (MAS) and ACE2 and to actively metabolize angiotensinogen (Bujak-Gizycka et al., 2019). Therefore, T47D cells represent a suitable model to elucidate the molecular targets sustaining the PM-mediated inflammation, and to highlight the possible role of PM as a booster of SARS-CoV-2-induced effects.

## Subsections

### The Renin-Angiotensin System and the Disrupting Activity of SARS-CoV-2

The RAS system orchestrates the delicate body fluids homeostasis and the complex balance of blood pressure through several receptors and their cognate ligands. The angiotensin converting enzyme (ACE) is the key-player in RAS system, converting angiotensin I in angiotensin II, which promotes vasoconstriction, inflammation, and reabsorption of sodium and water by binding AT1 receptor. The role of AT2 receptor is more elusive. It is highly expressed during fetal and early postnatal life, whilst its expression declines in adults. The AT2 receptor is considered to act as an antagonist of AT1-mediated effects, interplaying with ACE2.

The discovery of ACE2 in 2000 led to the reconsideration of roles and activities of the RAS proteins family.

ACE2 is a type I transmembrane protein, whose gene maps on chromosome X (Xp22.1). It presents a catalytically active ectodomain exposed to the circulation, which hydrolyzes several peptides. Its primary role is the cleavage of angiotensin I in the nonapeptide Ang 1-9, which binds AT2R, and angiotensin II in the heptapeptide Ang 1-7, binding MAS receptor. Through this enzymatic pathway of degradation, ACE2 coordinates the negative regulation of RAS and counteracts the adverse effects triggered by the binding of angiotensin I with AT1R (Patel et al., 2016). However, ACE2 can be hydrolyzed by the tumor necrosis factor converting enzyme (TACE), a disintegrin metalloprotease of the ADAM family (ADAM17), to a soluble form, which is released from the membrane. The ACE2 cleavage leads to the loss of ACE2 protection against RAS and increases the plasma levels of ACE2, which is considered a marker of risk for cardiovascular disease (Oudit et al., 2003).

In the subsequent years since its discovery, ACE2 was found to be able to degrade other substrates, including apelin-13, and dynorphin A 1-13 and to serve as the receptor for coronaviruses HCoV-NL63 and SARS-CoV (Hamming et al., 2008; Patel et al., 2016). The entry of coronaviruses and their ability to replicate of depends on the state of differentiation and polarization of epithelial cells and on the level of expression of ACE2 mRNA and protein (Jia et al., 2005).

It was first suggested and then confirmed that SARS-CoV-2 interacts with the ACE2 receptor for cellular internalization, and with TMPRSS2 serine protease for the Spike Protein cleavage (Hoffmann et al., 2020; Walls et al., 2020; Zhang et al., 2020). The new coronavirus S1 glycoprotein seems to be particularly optimized for the ACE2 cellular receptor, giving the virus a greater efficiency, in terms of tropism for the host (Walls et al., 2020). Interestingly, the SARS-CoV-2 S glycoprotein sequence shows a peculiar furin-like cleavage site, that is processed during biosynthesis, and has also been described for some highly pathogenic avian influenza viruses, although not for other coronaviruses (Coutard et al., 2020; Walls et al., 2020). The cellular entry of SARS-CoV-2 is facilitated by ADAM17 activity, which is upregulated by SARS-CoV through the cytoplasmatic domain of ACE2. The upregulation of ADAM17 perpetuates the loss of ACE2, leading to the disruption of the Ang 1-7/MAS receptor axis and accumulation of angiotensin II, which further upregulates ADAM17 with a positive feedback. Moreover, the upregulation of ADAM17 facilitates the liberation of membrane bound precursors of TNFα, IFN-γ, and IL-4 pro-inflammatory cytokines into the circulation, and as such, contributes to and sustains the cytokines storm (Wang et al., 2020).

### PM and the Receptors Interplaying in Adverse Outcomes by Chemical and Biological Agents

PM is a complex mixture whose composition varies according to the source of emission, and to season, geographical and meteorological factors. However it does typically show the same major components (sulfate, nitrate, ammonium, sodium and chloride, elemental carbon, organic carbon, mineral components, water, biological materials, carbon components), although in considerably different proportions according to the sampling location (Harrison and Yin, 2000). Airborne particles also contain minor components, such as metals and organic compounds, including PAHs. PM is always contaminated by PAHs generated from the incomplete combustion of organic material.

The aerodynamic diameter is the major determinant of the PM fate in the body. PM10 particles with an aerodynamic diameter ranging from 2.5 to 10 μm (inhalable particles) are deposited into the nasal cavities and the upper airways, whereas smaller particles, like fine (PM2.5) and ultrafine (PM0.1) particles (respirable particles), reach the bronchioles of the lung and then the alveoli. Fresh combustion-generated particles presumably retain their particle form after deposition, whereas secondary particles, including concentrated aqueous solutions, dissolve into the lung surfactant, delivering dissolved chemicals and sometimes releasing an ultrafine primary core. The release of chemicals is facilitated by the greater specific surface area of fine particles (Colacci and Vaccari, 2017).

Fine and ultrafine particles may cross the endothelial barrier, enter the blood stream and lead to adverse effects in the respiratory, cardiovascular, immune, and neural systems.

The adverse effects are strictly related to the concentrations of PM and its components. It has been demonstrated that the exposure-response relationship between PM2.5 and some adverse health outcomes, such as cardiovascular disease and mortality, is not linear, with a steep increase in risk at low exposure and flattening out at exposures above 50 μg/m3 (Pope et al., 2009). The association of PM2.5 exposure with lung cancer mortality, instead, appears to be nearly linear.

The toxicological properties increase linearly with increase in the PAHs concentration. Low concentrations trigger detoxification pathways, whilst higher concentrations activate an aberrant immune response, leading to genomic instability (Colacci et al., 2014; Vaccari et al., 2015; Mascolo et al., 2018; Serra et al., 2019). The entire process is orchestrated by the AhR.

The AhR is an evolutionary highly conserved, multifunctional cytosolic DNA-binding protein, working as a translocation factor, together with the AhR nuclear translocator (ARNT). The AhR is considered an environmental-sensor PAS protein, facilitating the entry and transport of small molecules.

It is the molecular target for dioxins, dioxin-like chemicals as well as PAHs, mediating the metabolic activation of xenobiotics and the response to environmental exposures. The complex AhR-ARNT binds specific DNA sequences, triggering the AhR signaling pathway, which leads to the transcription of the CYP1A1 and CYP1B1 genes, whose proteins play the main role in the bioactivation of several xenobiotics (Mascolo et al., 2018). The AhR is the only PAS protein that is activated by the ligand. Due to the different structures and properties of AhR ligands, AhR activation originates distinct biological effects. Over many years several studies and reviews have given further evidence for the intense crosstalk between AhR signaling and other signaling pathways, and genetic polymorphisms, starting with elucidation of the role of the AhR as a key bridge in cell and tissue physiology (Jacobs, 2005; Mulero-Navarro and Fernandez-Salguero, 2016). The role of AhR in the immune response and immune system homeostasis, which is related to some endogenous ligands, such as tryptophan, and the disrupting action of exogeneous ligands, has been previously described (Mascolo et al., 2018).

The ubiquitous distribution of the AhR in cells and tissues supports its implications in different physiological processes. During fetal development the AhR is highly expressed in lung, kidney, liver, and pancreas. In adulthood, high levels of AhR expression are detected in lung, placenta, spleen, and pancreas (Yi et al., 2018).

The presence of the AhR in heart and lung reinforces the recent discussion on the key role of the AhR in vascular physiological functions, including vascular development and angiogenesis as well as its involvement in vascular dysfunction and diseases. The AhR has been described as playing a role in myocarditis, hypertension, atherosclerosis, ischemic heart disease, and pulmonary arterial hypertension (Mulero-Navarro and Fernandez-Salguero, 2016; Yi et al., 2018). Recent reports suggest an interplay between the AhR and angiotensin II, through the crosstalk of AhR and PPARalpha signaling pathways (Ichihara et al., 2019). An interplay between the AhR and MAS receptors has been also described in response to uremic toxins (Ng et al., 2016). These reports support the hypothesis that the activated RAS requires the integrity of the AhR to maintain normal blood pressure (Zhang et al., 2010).

Therefore, the AhR can be postulated to be the key bridging receptor in mediating the effects elicited by PM on cardiovascular system.

The PM interference with the RAS system has been well documented. Several studies in mice and rats have correlated the exposure to PM with the up-regulation of the Ace and At1r genes and proteins at the lung tissue level inducing a simultaneous overproduction of inflammatory molecules, including IL-6 (Aztatzi-Aguilar et al., 2015; Lin et al., 2018; Sharma et al., 2019).

The pro-inflammation angiotensin II-activated pathway MAPK (ERK 1/2, p38)-STAT3, is upregulated in mice after PM 2.5 exposure. In ACE2 knockout mice, the exposure to PM 2.5 increases the production of IL-6, tumor necrosis factor alpha (TNF-α) and tumor growth factor beta 1 (TGF-β1), which is linked to the prolonged activation of the MAPK(ERK 1/2, p38)-STAT3 pathway, as confirmed by the increased levels of p-ERK1/2 and p-STAT3 levels (Lin et al., 2018).

In addition to the common mode of action related to RAS perturbation, another interesting key event seems to emerge, upstream in the adverse outcome pathway leading to cardiovascular disease. Several recent studies suggest the involvement of the endoplasmic reticulum (ER) in the mechanisms sustaining vascular endothelial dysfunctions. An ER-mediated molecular mechanism has been described, starting with ER instability, leading to the activation of IRE1α/XBP1s branch of the unfolded protein response (UPR), that affects HIF1α transactivation to mediate angiotensin II-dependent endothelial dysfunction (Xu et al., 2017). ER stress is caused by both PM and viral infections (Watterson et al., 2009; Chan, 2014; Xu et al., 2017).

Global proteomic and microarray analyses in particular have shown the up-regulation of several genes related to the ER stress in cells infected with SARS-CoV. The coronavirus-induced ER stress can be explained by: (i) massive morphological rearrangement of the ER; (ii) significant increase ER burden for protein synthesis, folding and modification; and (iii) extensive depletion of ER lipid component (Fung and Liu, 2014). The coronavirus E (envelope) protein has been demonstrated to modulate the IRE1-XB1 pathway (DeDiego et al., 2011).

## Molecular Pathways of Inflammatory Effects Caused by Cell Exposure to PM: An Original Experimental Study

### The Experimental Design

**FIGURE 1:**
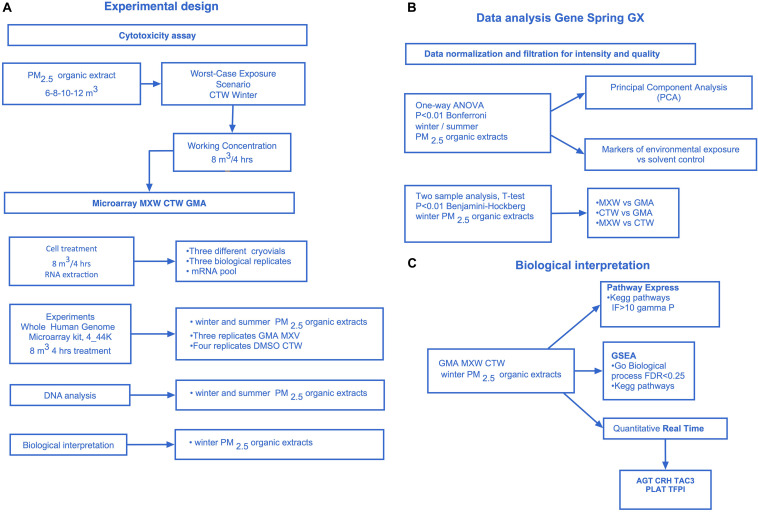
Experimental design. **(A)** The experimental design. T47D Cytotoxicity assay, Microarray experiments (whole human genome, 4 × 44k): winter and summer PM_2__.__5_ extracts 8 m^3^ 4 h treatment, Data analysis: winter and/or summer PM_2__.__5_ extracts, Biological interpretation: winter PM_2__.__5_ extracts. **(B)** GeneSpring (GeneSpring GX, Agilent Technologies) Data analysis. Data normalization and filtration for intensity and quality signal, One-Way ANOVA Analysis (winter and summer PM_2__.__5_ extracts, *P* < 0.01 Bonferroni, 11,483 differentially expressed genes) to select differentially expressed genes among the treatments and the control, Principal Component Analysis (PCA), Analysis of markers of environmental exposure (CYP1A1, CYP1B1, HMOX1), *t*-test analysis on winter PM_2__.__5_ samples: MXW vs. GMA, CTW vs. GMA, MXW vs. CTW (*P* < 0.01 Benjamini–Hockberg). **(C)** Biological interpretation on winter PM_2__.__5_ extracts. Microarray analysis: Pathway express (PE), Gene Set Enrichment Analysis (GSEA). Real time PCR: AGT, CRH, TAC3, PLAT, TFPI.

Figure 1 shows the experimental design and the steps of the experimental protocol.

### Collection of the Samples and Cell Treatment

PM2.5 samples were collected during summer 2008 and winter 2009 at several sites located in the surroundings of Bologna (Emilia Romagna, Italy), chosen on the basis of air dispersion models and representative of different levels of environmental pollution. They included: (1) a site typical of the urban background (GMA 25.96 μg/m3), (2) a site affected by the maximum fall-out of the emission of a waste-to-energy plant (MXW 35.42 μg/m3), which was chosen as a punctual source, (3) a site that was considered the minimum fall-out point of the punctual source (CTW 34.47 μg/m3), but was affected by all the other emission sources in the area, including heavy traffic. For further details see Supplementary Table S1. The organic fractions, obtained by an acetone extraction, were reduced to dryness and then dissolved in DMSO at a final concentration of 800 m3 equivalents/ml. The treatment concentrations were chosen taking into account three parameters: (1) the need of simulating a realistic human exposure, (2) the level of concentration that was not cytotoxic, and (3) the level of concentration ensuring the cell response at the gene level. Based on these considerations, a preliminary cytotoxicity test was performed exposing cells to a range of concentrations. The top concentration 12 m3 (414 μg/plate/48 h exposure time) represented the highest exposure for residents in the Po Valley in 12 h/day of outside activity. The lowest experimental concentration was representative of the infant exposure that we calculated as being 0.9 m3/day. This calculation was performed for the worst-case exposure scenario, that is, the most polluted site, in the most polluted season (e.g., samples from CTW collected in winter) (Supplementary Figure S1 and Supplementary Table S2). The gene expression analysis experiments were designed to compare the effects of equal volumes of inhaled air from different exposure scenarios, by choosing the same volumetric concentration for all the tested samples, even if the amount of PM was different (Supplementary Table S1). Therefore, the dose of the organic extracts corresponding to 8 m^3^ of filtered air volume was chosen as the treatment concentration for all the PM samples. Several literature reports have shown that it is possible to measure the transcriptional response in cells exposed to complex environmental mixtures, after 4–6 h (Maunders et al., 2007; Sen et al., 2007). Moreover, preliminary experiments had showed the strong induction of CYP1A1, the key enzyme for the metabolism of several xenobiotics and the main marker of the AhR-mediated pathway, in T47D cells after 4 h-exposure to dioxin-like chemicals and PAHs.

Cells were exposed for a period of 4 h, according to the need to simulate an average daily exposure in outside environments, and to ensure the cell response at the molecular level.

Appropriate negative (untreated cells) and solvent (DMSO)-treated cell controls were also carried out.

### Microarray Data Analysis

The PCA on one-way analysis of variance (One-Way ANOVA, *p* < 0.01, Bonferroni; 11,483 differentially expressed genes) showed that summer extracts clustered with the controls, confirming the seasonal influence on the biological response with the winter extracts representing the worst case exposure scenario. Moreover, MXV and CTW winter samples cluster together and far from controls and GMA. The results from Hierarchical Clustering approach highlighted a similar behavior (Supplementary Figure S2).

Well-known markers of environmental exposures, CYP1A1, CYP1B1 and HMOX1, were upregulated after T47D treatment with PM2.5 (Supplementary Figure S3) supporting the choice of exposure time.

Taking into account the seasonal effect on transcription, the additional analysis specifically focused on the winter samples as representative of the worst exposure scenario.

The *t*-test analysis was applied to compare the transcriptional profiles of the three sites (MXW vs. GMA, CTW vs. GMA, MXW vs. CTW) and identify the corresponding gene modulations induced by each treatment. The comparison MXW vs. CTW returned the lower number of differentially expressed genes (DEGs), suggesting that these two sites differed less from each other than from the urban background site GMA (Supplementary Table S3).

### Biological Interpretation

The biological interpretation was performed by using Pathway Express (PE Intelligent Systems and Bioinformatics Laboratory), and by applying a Gene Set Enrichment Analysis (GSEA). Real-time PCR was carried out to confirm microarray modulation of a specific gene set.

**FIGURE 2:**
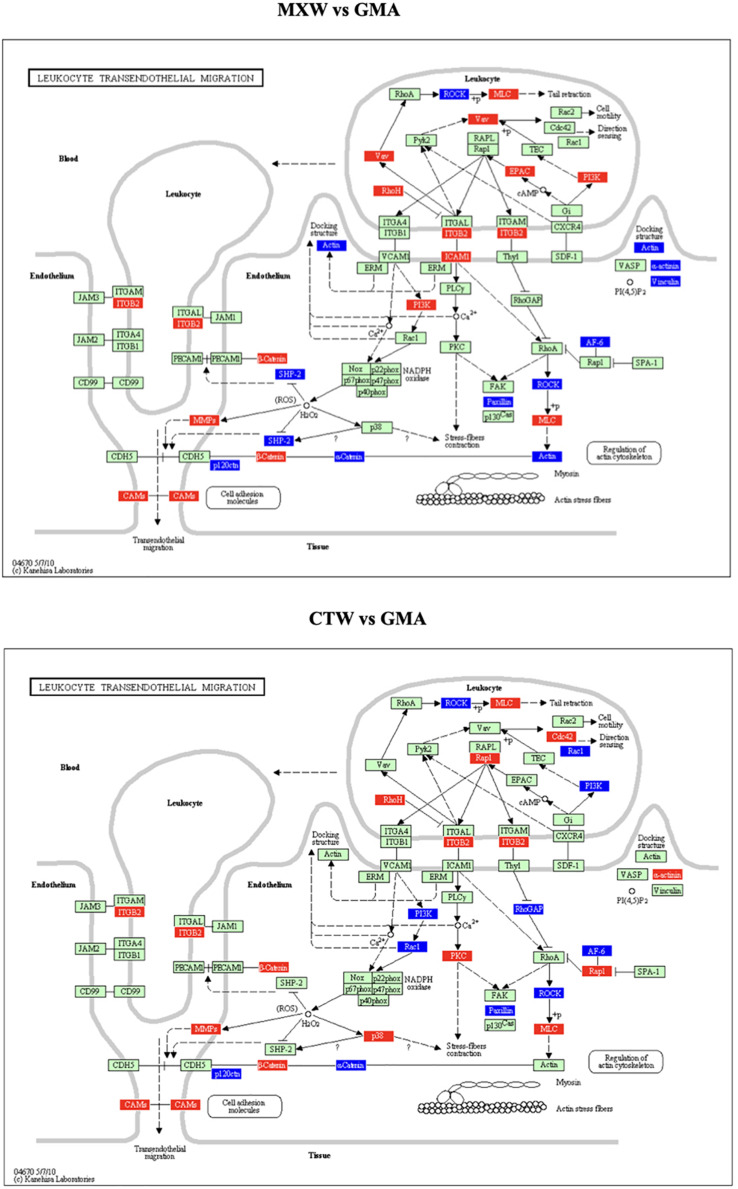
Leukocyte transendothelial migration KEGG, as obtained from Pathway Express (PE) analysis, MXW vs. GMA, CTW vs. GMA. Red: up-regulation. Blue: down-regulation.

In Table 1 the statistically significant KEGGs pathways obtained by the PE analysis are reported. The KEGG pathway “Cell Adhesion Molecules (CMAs),” which includes the “Leukocyte Transendothelial Migration” and the “Adherence Junction” pathways, was affected in both comparisons. The gene modulations of the “Leukocyte Transendothelial Migration” KEGG pathway in the comparisons MXW vs. GMA and CTW vs. GMA showed a similar pattern of up or down regulation of components of cytoskeleton and adhesion systems (Figure 2).

**TABLE 1:** Pathway express analysis – list of KEGG pathways, ranked for the impact factor value, that resulted transcriptionally perturbated (Impact Factor IF > 10, gamma-*p* value < 0.05).

Winter campaign					
MXW vs. GMA		CTW vs. GMA		MXW vs. CTW	
1	Leukocyte transendothelial migration	1	Antigen processing and presentation	1	Cell adhesion molecules (CAMs)
2	Cell adhesion molecules (CAMs)	2	Leukocyte transendothelial migration	2	Leukocyte transendothelial migration
3	Antigen processing and presentation	3	Cell adhesion molecules (CAMs)	3	Adherens junction
4	Adherens junction	4	Adherens junction	4	Phosphatidylinositol signaling system
5	Circadian rhythm	5	Phosphatidylinositol signaling system		
6	Phosphatidylinositol signaling system	6	Circadian rhythm		

The modulation of the strength and extent of cell-cell and cell-substratum adhesion is highly relevant in cellular processes requiring major changes in plasticity. The deregulation of cytoskeletal components and the disturbance of the adhesion systems is associated with the acquisition of the locomotor phenotype and the increased cell motility (Colacci et al., 2014).

Neither the innate nor the adaptive immune system “responds” unless leukocytes cross blood vessels. The process of leukocyte extravasation, a critical step in the inflammatory/immune response, involves the migration of leukocytes from the bloodstream towards target tissues, where they exert their effector function (Barreiro and Sánchez-Madrid, 2009).

Neutrophil recruitment is a multistep process, which includes capture/attachment, rolling, firm arrest, spreading, and extravasation/migration. Each step requires crosstalk between neutrophils and endothelial cells to orchestrate this dynamic phenomenon (Kilpatrick and Kiani, 2020).

Therefore, transendothelial migration (TEM) is considered to be the committed step and a point of no return in the inflammatory response.

Leukocyte extravasation is orchestrated by the coordinated action of cellular adhesion receptors, chemotactic factors, cytoskeleton, signaling molecules and involves radical morphological changes in both leukocytes and endothelial cells. This “active” process for both cell types promotes the rapid and efficient influx of leukocytes to inflammatory foci without compromising the integrity of the endothelial barrier.

Several endothelial cell molecules, such as intracellular adhesion molecules (ICAM-1), vascular cell adhesion molecules (VCAM-1), junctional adhesion molecules (JAMs), endothelial cell-selective adhesion molecule (ESAM), platelet endothelial cell adhesion molecule-1 (PECAM-1), poliovirus receptor (PVR), CD99 and CD99L2, support TEM in a sequential fashion (Masgrau-Alsina et al., 2020).

The deregulation of adhesion molecules and cytoskeleton components, following PM2.5, cell exposure is depicted in Figure 2.

When properly contained, inflammation leads to the recruitment and activation of circulating leukocytes to restore tissue and organism homeostasis. Although this process is critical for successful wound healing and the elimination of pathogens and infections, misdirected inflammation can exacerbate pathology and cause substantial morbidity and mortality. Inflammation is a fundamental process that underlies the pathology of virtually all diseases including arthritis, atherosclerosis, and multiple sclerosis (Sullivan et al., 2020), and non-genotoxic carcinogenicity (Jacobs et al., 2020).

The GSEA results demonstrated a general positive enrichment in KEGG pathways and GO biological processes triggered by CTW and MXW extracts with respect to GMA. The analysis did not give evidence for any significant enrichment when comparing MXW with CTW, further confirming that the transcriptional effect induced by MXW or CTW samples was quite similar (Supplementary Table S4).

In Tables 2, 3 the complete list of enriched (FDR < 0.25) KEGG pathways and GO biological processes is reported.

**TABLE 2:** GSEA analysis – lists of KEGG pathways, ranked for the Enrichment Score, significantly enriched (FDR < 0.25) in MXW vs. GMA, CTW vs. GMA.

	Enriched in MXW vs. GMA		Enriched in CTW vs. GMA
1	Cytokine_cytokine_receptor_interaction	1	Systemic_lupus_erythematosus
2	Intestinal_immune_network_for_Iga_production	2	Ribosome
3	Graft_versus_host_disease	3	Cytokine_cytokine_receptor_interaction
4	Complement_and_coagulation_cascades	4	Hematopoietic_cell_lineage
5	Asthma	5	Intestinal_immune_network_for_Iga_production
6	Arachidonic_acid_metabolism	6	Complement_and_coagulation_cascades
7	Histidine_metabolism	7	Graft_versus_host_disease
8	Hematopoietic_cell_lineage	8	Asthma
9	Ether_lipid_metabolism	9	Allograft_rejection
10	Cell_adhesion_molecules_cams	10	Type_I_diabetes_mellitus
11	Autoimmune_thyroid_disease	11	Tgf_beta_signaling_pathway
12	Type_I_diabetes_mellitus	12	Antigen_processing_and_presentation
13	Antigen_processing_and_presentation	13	Arachidonic_acid_metabolism
14	Glycine_serine_and_threonine_metabolism	14	Nod_like_receptor_signaling_pathway
15	Allograft_rejection	15	Autoimmune_thyroid_disease
16	Systemic_lupus_erythematosus	16	Oxidative_phosphorylation
17	Hedgehog_signaling_pathway	17	Vegf_signaling_pathway
18	Nod_Iike_receptor_signaling_pathway	18	Bladder_cancer
19	Leishmania_infection	19	Ether_Lipid_Metabolism
20	Beta_alanine_metabolism		
21	Tgf_beta_signaling_pathway		
22	Primary_immunodeficiency		
23	Snare_interactions_in_vesicular_transport		
24	Long_term_depression		
25	Leukocyte_transendothelial_migration		
26	Vascular_smooth_muscle_contraction		
27	Arginine_and_proline_metabolism		
28	Drug_metabolism_cytochrome_p450		
29	Viral_myocarditis		
30	Glycerophospholipid_metabolism		
31	Basal_cell_carcinoma		

**TABLE 3:** GSEA analysis – lists of GO-Biological Processes, ranked for the Enrichment Score, significantly enriched (FDR < 0.25) in MXW vs. GMA, CTW vs. GMA.

	Enriched in MXW vs. GMA		Enriched in CTW vs. GMA
1	Wound_healing	1	Wound_healing
2	Hemostasis	2	Regulation_of_multicellular_organismal_process
3	Coagulation	3	Regulation_of_growth
4	Blood_coagulation	4	Adaptive_immune_response
5	Regulation_of_body_fluid_levels	5	Negative_regulation_of_growth
6	Regulation_of_multicellular_organismal_process	6	Coagulation
7	Female_pregnancy	7	Blood_coagulation
8	Negative_regulation_of_growth	8	Positive_regulation_of_immune_system_process
9	Immune_response	9	Humoral_inunune_response
10	Regulation_of_response_to_stimulus	10	Hemostasis
11	Regulation_of_growth	11	Female_pregnancy
12	Growth	12	Regulation_of_immune_system_process
13	Response_to_external_stimulus	13	Immune_response
14	Behavior	14	Growth
15	Immune_system_process	15	Negative_regulation_of_dna_binding
16	Axonogenesis	16	Regulation_of_T_cell_activation
17	Response_to_wounding	17	Negative_regulation_of_binding
18	Regulation_of_cell_growth	18	Regulation_of_cell_growth
19	Negative_regulation_of_dna_binding	19	Immune_system_process
20	Cell_cell_signaling	20	Regulation_of_lymphocyte_activation
21	Humoral_immune_response	21	Positive_regulation_of_cytokine_biosynthetic_process
22	Adaptive_immune_response	22	Generation_of_a_sigual_involved_in_cell_cell_signaling
23	Cell_Surface_Receptor_Linked_Signal_Trasduction_process	23	Positive_regulation_of_multicellular_organismal_process
		24	Regulation_of_muscle_contraction
		25	Adaptive_immune_response _go_0002460
		26	Response_to_external_stimulus
		27	Positive_regulation_of_translation
		28	Reproductive_process
		29	Locomotory_behavior

**FIGURE 3:**
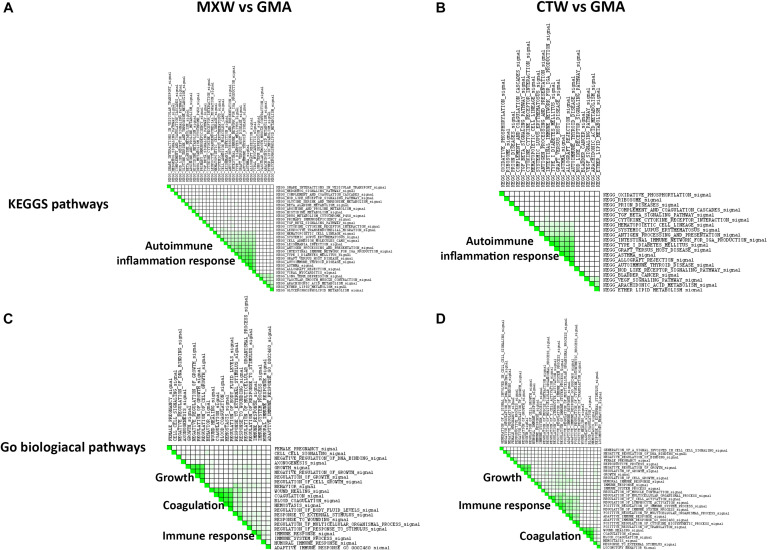
GSEA leading edge analysis of the significantly enriched KEGG pathway **(A,B)** and GO Biological Processes **(C,D)**. The results are shown as a similarity matrix where the intensity of the green color directly correlates with the extent of the intersection between the leading edge core genes of each gene set combination. **(A,C)** = MXW vs. GMA; **(B,D)** = CTW vs. GMA.

The leading edge analysis applied on the list of the enriched KEGG pathway gene sets showed the activation of immune response and inflammation as the main biological themes enriched in MXW and CTW (Figure 3). The enrichment of several pathways involved in the immune and autoimmune response, such as “Systemic lupus erythematosus,” “Intestinal immune network for IgA production” or “Graft versus host disease,” was sustained by the up-regulation of genes encoding for CD86, a protein expressed on antigen-presenting cells necessary for T cell activation and survival, and by the transcriptional modulation of HLA genes belonging to Major Histocompatibility Class II (MHC II), some of which are involved in the etiology of the autoimmune disease. The transcriptional activation of the genes encoding for enzymes such as arachidonate 5-lipoxygenase (ALOX5) and phospholipase A2 group III (PLA2G3), accounted for the enrichment of the other small groups of pathways, such as “Arachidonic acid metabolism,” that, in addition to lipid metabolic pathways, are involved in the synthesis of important mediators of the inflammatory response.

In the list of the enriched KEGG pathway gene sets “Type 1-diabetes mellitus” also appeared, Type 1- diabetes is an autoimmune condition resulting from the autoimmune destruction of the insulin-producing beta-cells. Certain beta-cell proteins act as autoantigens after being processed by antigen-presenting cells (APC), such as macrophages and dendritic cells, and presented in a complex with MHC-II molecules on the surface of the APC. Then, immunogenic signals from APC activate CD4+ T cells, predominantly of the Th1 subset. Antigen-activated Th1 cells produce IL-2 and IFNgamma. They activate macrophages and cytotoxic CD8+ T cells, and these effector cells may kill islet beta-cells by one or both of two types of mechanisms: (1) direct interactions of antigen-specific cytotoxic T cells with a beta-cell autoantigen-MHC-I complex on the beta-cell, and (2) non-specific inflammatory mediators, such as free radicals/oxidants and cytokines (IL-1, TNFalpha, TNFbeta, IFNgamma). Type I diabetes is a polygenic disease and one of the principle determining genetic factors in diabetes incidence is the inheritance of mutant MHC-II alleles.

In the comparison of MXV vs. GMA, we observed the modulation of genes involved in this KEGG pathway, such as GAD1 and several HLA genes. GAD1, which when upregulated, encodes for one of several forms of glutamic acid decarboxylase. This enzyme has a pathogenic role in the human pancreas and is identified as a major autoantigen and autoreactive T cell target in insulin-dependent diabetes.

The GSEA leading edge analysis allowed identification of “Coagulation,” “Growth Control,” and “Immune Response” as the three major GO Biological Processes in both the comparison, MXW vs. GMA and CTW vs. GMA (Figure 3). Cells treated with CTW or MXW showed a similar, even if not identical, pattern with a higher enrichment score in MXW-treated samples for the commonly modulated processes (Table 3). For example, the coagulation process (“Blood Coagulation” GO: 0007596) and its associated GO terms showed a higher enrichment score in MXW. A similar behavior can also be observed for “Female Pregnancy” (GO: 0007565).

The main genes involved in the “Blood Coagulation” GO process, which were enriched in both MXW vs. GMA and CTW vs. GMA comparisons, were PLAT and TFPI (Table 4). These genes sustained also the enrichment scores for the “Wound Healing” biological process.

**TABLE 4:** Lists of the GSEA leading edge core genes that account for the enrichment of the GO biological process “Blood Coagulation” (GO: 0007596).

MXW vs. GMA		CTW vs. GMA	
GSEA NES 2.l FDR 0.003; nominal *p*-value < 0.01		GSEA NES 1.74 FDR 0.146;nominal *p*-value < 0.01	
PLAT	Plasminogen activator, tissue type	PLAT	Plasminogen activator, tissue type
F7	Coagulation factor VII	TFPl	Tissue factor pathway inhibitor
TFPl	Tissue factor pathway inhibitor	PF4	Platelet factor 4
GNAQ	G protein subunit alpha q	F7	Coagulation factor VII
PF4	Platelet factor 4	F12	Coagulation factor XI I
F2	Coagulation factor II, thrombin	ITGA2	Integrin subunit alpha 2
LMAN1	Lectin, mannose-binding 1	F2	Coagulation factor II, thrombin
F5	Coagulation factor V	PROSl	Protein S
PLG	Plasminogen	PLG	Plasminogen
F12	Coagulation factor XI I	WAS	WASP actin nucleation promoting factor
WAS	WASP actin nucleation promoting factor		
C4BPB	Complement component 4 binding protein beta		
TMPRSS6	Transmembrane serine protease 6		

*Normalized Enrichment score (NES), FDR and nominal p-value were reported for each comparison.*

The PLAT gene encodes for the tissue-type plasminogen activator (tPA), a secreted serine protease that converts the proenzyme plasminogen to plasmin. The protein is a fibrinolytic enzyme involved in the breakdown of blood clots (Yu et al., 2019). Increased enzymatic activity causes hyperfibrinolysis, which develops into excessive bleeding.

Endothelial expression of tissue-type plasminogen activator is crucial for maintaining the adequate endogenous fibrinolysis (Glise et al., 2019). Along with ADAMTS1 and PLAU and TIMP3, PLAT gene results upregulated in severe wall shear stress (WSS), are due to chronic high flow that can induce arterial remodeling (Dolan et al., 2012).

It has been reported that in an *in vitro* cell system of bovine oocytes, the tissue type plasminogen activator and its inhibitor serpin family E member 1 (Serpine1) cooperatively regulate PLAT activity in various reproductive processes (Yu et al., 2019). Moreover, in the *in vitro* fertilization (IVF) “superovulation” process, the process is frequently associated with an increased risk of disorders of placentation such as preeclampsia and fetal growth restriction (FGR), and alteration of gene expression, such as PLAT, critical to endometrial remodeling during early implantation. Such changes could lead to altered trophoblast migration and impaired endovascular invasion (Senapati et al., 2018). PLAT is also one of the 16 genes composing the “associative network” connecting preeclampsia with diabetes mellitus, gestational diabetes and obesity, which are generally concurrent pathologies (Glotov et al., 2015).

In addition, the astrocyte plasminogen activator system may play a major role in the modulation of neuronal plasticity. Ethanol-induced upregulation of tPA levels, encoded by the gene PLAT, and plasmin activity may be responsible for altered neuronal plasticity in fetal alcohol spectrum disorders (FASD) (Wilhelm et al., 2018).

The product of the gene TFPI is a Kunitz-type serine protease inhibitor that regulates the tissue factor (TF)-dependent pathway of blood coagulation, inhibiting the activated factor X and VIIa-TF proteases in an autoregulatory loop. The product of TFPI gene is an anticoagulant protein that is expressed primarily in the vascular endothelium, megakaryocytes, platelets and plasma (Naji et al., 2018).

TFPI was reported to reduce the development of atherosclerosis by regulating tissue factor (TF)-mediated coagulation pathway, exerting an inhibitory effect on endothelial cell activation, vascular smooth muscle cell (VSMC) proliferation and migration, inflammatory cell recruitment and extracellular matrix, all of which are associated with the development of atherosclerosis (Yuan et al., 2019).

Additionally, a genomic TFPI variant is significantly associated with fibrinogen levels and risk of coronary artery disease (CAD) (Naji et al., 2018).

Patients with early onset of preeclampsia (EOP), characterized by competing thrombotic and bleeding risks, show an attenuated coagulation response with reduced thrombin generation stimulated by low-dose TF and elevated activity of plasma TFPI (Egan et al., 2017).

TFPI-2 was remarkably up-regulated in both serum and placenta of patients affected by preeclampsia, closely associated with trophoblast cell dysfunction. It was reported that hypoxia/reoxygenation, that mimics the oxidative stress state of preeclampsia, increased the expression of TFPI-2 in a HTR-8/SVneo cell line used to simulate the primary trophoblast cells (Zheng et al., 2020).

Finally, the list of differentially expressed genes strictly related to the PM samples from MGW includes apelin and ADAM17 (TACE), which are upregulated, confirming the involvement of RAS and transmembrane disintegrins in the response to PM treatment (Table 5).

**TABLE 5:** List of differentially expressed genes MXW vs. GMA.

Gene symbol	Gene name	D.E.
AGT	Angiotensinogen	Up
TFPI	Tissue factor pathway inhibitor	Up
CRH	Corticotropin releasing hormone	Up
PLAT	Plasminogen activator, tissue type	Up
TAC3	Tachykinin precursor 3	Up
APLN	Apelin	Up
ADAM 17 (TACE)	ADAM metallopeptidase domain 17	Up
IL18BP	Interleukin 18 binding protein	Up
IL18	Interleukin 18	Down
IL27	Interleukin 27	Down
IL23A	Interleukin 23 subunit alpha	Up
IL1F10	Interleukin 1 family member 10	Up
IL11	Interleukin 11	Up
IL17D	Interleukin 17D	Up
IL32	Interleukin 32	Up
IL22RA1	Interleukin 22 receptor subunit alpha 1	Up
IL3RA	Interleukin 3 receptor subunit alpha	Up
IL13A1	Interleukin 13 receptor subunit alpha 1	Up
IL1R1	Interleukin 1 receptor type 1	Up
IL27RA	Interleukin 27 receptor subunit alpha	Up
CCR7C-C	C-C motif chemokine receptor 7	Up
CCR10	C-C motif chemokine receptor 10	Up
CX3CL1	C-X3-C motif chemokine ligand 1	Up
CKLF	Chemokine like factor	Up
CXCL12	C-X-C motif chemokine ligand 12	Up
CXCL3	C-X-C motif chemokine ligand 3	Up
CCL17	C-C motif chemokine ligand 17	Up
CCL21	C-C motif chemokine ligand 21	Up
CCL28	C-C motif chemokine ligand 28	Up
CCL2	C-C motif chemokine ligand 2	Up
CCL3	C-C motif chemokine ligand 3	Up
HCG9	HLA complex group 9	Up
HLA-DMB	Major histocompatibility complex, class II, DM beta	Up
HLA-DRB5	Major histocompatibility complex, class II, DR beta 5	Up
HLA-DRB4	Major histocompatibility complex, class II, DR beta 4	Up
HLA-DRB3	Major histocompatibility complex, class II, DR beta 3	Up
COX18	Cytochrome c oxidase assembly factor COX18	Up
SULT1A2	Sulfotransferase family 1A member 2	Up
EDN2	Endothelin 2	Up
EFNA1	Ephrin A1	Up
NOS3	Nitric oxide synthase 3	Up
HIF1A	Hypoxia inducible factor 1 subunit alpha	Up
PSG7	Pregnancy specific beta-1-glycoprotein 7	Up
PSG9	Pregnancy specific beta-1-glycoprotein 9	Up
PSG4	Pregnancy specific beta-1-glycoprotein 4	Up
PSG1	Pregnancy specific beta-1-glycoprotein 1	Up
PSG8	Pregnancy specific beta-1-glycoprotein 8	Up
PSG6	Pregnancy specific beta-1-glycoprotein 6	Up
PSG11	Pregnancy specific beta-1-glycoprotein 11	Up
PSG5	Pregnancy specific beta-1-glycoprotein 5	Up

### The Molecular Signature of Adverse Reproductive Outcomes

It is noteworthy that homeostatic leukocyte trafficking into and within the female reproductive tract (FRT) contributes to fertility and reproductive health. It is unclear how this process is regulated in the anatomically distinct reproductive tissues, or whether the genes involved are affected by cyclical changes in reproductive hormones (Menzies et al., 2020). Eutherian embryo implantation is evolutionarily derived from and homologous to a defensive inflammatory process. The inflammatory response is inducted, maintained and strictly regulated. throughout the pregnancy, due to the recruitment of different populations of maternal leucocytes, which mark the different stages of gestation. In mammals, inflammation is necessary in implantation and parturition and it is sustained by cytokines (IL-6, IL-8, TNFα) secreted by uterine-specific natural killer (uNK) cells, which represent 70% of cells during the first trimester, and by neutrophils, which are recruited at term. After implantation, the inflammatory response is temporarily suppressed whilst a fetal-maternal communication network emerges, by shifting the inflammatory cell-cell communication network to a different set of cell types, with monocytes and macrophages differentiating to the predominant M2 alternative activation profile and exhibiting anti-inflammatory behavior (Ramhorst et al., 2019).

Therefore, the ancestral inflammatory reaction to the embryo attachment evolved into a cooperative fetal-maternal communication network (cooperative inflammation) (Stadtmauer and Wagner, 2020).

The “Female Pregnancy” GO process includes genes that are involved in the different steps of gestation, from fertilization to birth (Table 6). Among them, angiotensinogen gene (AGT), corticotropin releasing hormone (CRH) tachykinin 3 (TAC3) were upregulated and drove the enrichment scores.

**TABLE 6:** List of the GSEA leading edge core genes that account for the enrichment of the GO biological process “Female Pregnancy” (GO: 0007565).

MXW vs. GMA		CTW vs. GMA		MXW vs. CTW	
NES 1.9 FDR 0.04 nominal *p*-value < 0.01		NES 1.68 FDR 0.17 nominal *p*-value < 0.01		NES 1.72 FDR 1 nominal *p*-value < 0.01	
AGT	Angiotensinogen	AGT	Angiotensinogen	HPGD	15-hydroxyprostaglandin dehydrogenase
CRH	Corticotropin releasing hormone	CSH2	Chorionic somatomammotropin hormone 2	CRH	Corticotropin releasing hormone
TAC3	Tachykinin precursor 3	TAC3	Tachykinin precursor 3	PSG7	Pregnancy specific beta-1-glycoprotein 7
HPGD	15-hydroxyprostaglandin dehydrogenase	PPARD	Peroxisome proliferator activated receptor delta	PSG5	Pregnancy specific beta-1-glycoprotein 5
COL16A1	Collagen type XVI alpha 1 chain	CRH	Corticotropin releasing hormone	PSG4	Pregnancy specific beta-1-glycoprotein 4
PSG5	Pregnancy specific beta-1-glycoprotein 5	SPRR2D	Small proline rich protein 2D	COL16A1	Collagen type XVI alpha 1 chain
PSG7	Pregnancy specific beta-1-glycoprotein 7	COL16A1	Collagen type XVI alpha 1 chain	AGT	Angiotensinogen
PSG9	Pregnancy specific beta-1-glycoprotein 9	PRLHR	Prolactin releasing hormone receptor	PSG11	Pregnancy specific beta-1-glycoprotein 11
GHRL	Ghrelin and obestatin prepropeptide	ADM	Adrenomedullin	PSG8	Pregnancy specific beta-1-glycoprotein 8
CSH2	Chorionic somatomammotropin hormone 2			TAC3	Tachykinin precursor 3
ADM	Adrenomedullin			SCGB1A1	Secretoglobin family 1A member 1
FCGRT	Fc fragment of IgG receptor and transporter			PSG6	Pregnancy specific beta-1-glycoprotein 6
TFCP2L1	Transcription factor CP2 like 1			PSG1	Pregnancy specific beta-1-glycoprotein 1
PSG8	Pregnancy specific beta-1-glycoprotein 8			PSG9	Pregnancy specific beta-1-glycoprotein 9
PSG1	Pregnancy specific beta-1-glycoprotein 1				
SCGB1A1	Secretoglobin family 1A member 1				
PSG6	Pregnancy specific beta-1-glycoprotein 6				
PSG11	Pregnancy specific beta-1-glycoprotein 11				
GHSR	Growth hormone secretagogue receptor				
SPRR2D	Small proline rich protein 2D				

*Normalized Enrichment Score (NES), FDR and nominal *p*-value were reported for each comparison.*

Angiotensinogen gene is the precursor of angiotensin I. Increased expression of AGT was demonstrated to be associated with the development and progression of bronchopulmonary dysplasia (BPD) originated from persistent ER stress. In A549 cell system AGT overexpression resulted in the inflammation via the JAK/STAT signal pathway (Shen et al., 2017). Similarly, AGT which is modulated by binding to miR-149-5p, is reported to promote the IL-6-induced inflammatory responses of chondrocytes in osteoarthritis (OA) via JAK2/STAT3 pathway (Wang et al., 2020).

Renin-angiotensin axis is also a critical regulator of placental function, controlling trophoblast proliferation, angiogenesis and blood flow. RAS, which significantly influences uteroplacental blood flow through the balance of its vasoconstrictive and vasodilatory pathways, is dysregulated in placentae from women with preeclampsia. This dysregulation could lead to the reduced placental perfusion that is evident in FGR (Delforce et al., 2019).

It has been reported that there is a relationship between genetic variations in AGT and AGTR1, and the influence of RAS on the onset of preeclampsia and idiopathic recurrent pregnancy (Heidari et al., 2019; Procopciuc et al., 2019). For pregnant women with gestational hypertension or preeclampsia, they are also known to have inappropriate intrarenal renin–angiotensin system activation, increased urinary excretion of AGT and potassium associated with signs of glomerular swelling (Shono et al., 2019).

Corticotropin releasing hormone encodes for a hypothalamic peptide, the Corticotropin-releasing hormone well-known as the central hormone of the hypothalamic–pituitary–adrenocortical (HPA) axis, a critical neuroendocrine system regulating responses to stressful stimuli (Raftogianni et al., 2018).

Elevated CRH serum concentrations, as compared with gestational age matched controls, occur in patients in preterm labor. Maternal plasma CRH levels increase exponentially as pregnancy advances, peaking at the time of delivery. Women delivering preterm have increased levels of CRH early in pregnancy, while those who deliver post-term tend to have lower CRH levels (Torricelli et al., 2011; Wang et al., 2018). Elevated serum CRH level has been linked to a number of adverse pregnancy outcomes including preeclampsia, FGR and gestational diabetes mellitus (Wang et al., 2018).

Placental CRH is considered to play a crucial role in the regulation of fetal maturation and the timing of delivery as well as in the control of fetal-placental blood flow (You et al., 2012; Stamatelou et al., 2013). Placental CRH has been proposed to be part of the mechanism that governs the length of gestation in humans, contributing, in concert with other factors, to the regulation of a placental clock to modulate a cascade of physiological events leading to parturition (Wang et al., 2018). In addition to maturation of the human fetus, placental CRH has been associated with postnatal child behavioral and brain development (Ramos et al., 2019).

Dysregulation of the hypothalamic pituitary adrenal (HPA) axis, has been also related to mood disorders, including postpartum depression (PPD2). Transcriptional control of CRH by nuclear steroid hormone receptors has been described as a key mechanism for regulating CRH production and preventing the excessive activity of HPA axis (Zoubovsky et al., 2020).

TAC3 is a member of the tachykinin family. The gene product, neurokinin B (NKB), is mainly expressed in the central and peripheral nervous system where it acts as a neurotransmitter. NKB is synthesized in discrete neuronal populations within the hypothalamus, where it participates in the regulation of gonadotropin-releasing hormone (GnRH) secretion. Neurokinin B (NKB) and its receptor, NK3R, play critical roles in reproduction by regulating the secretion of the hypothalamic GnRH (Qi et al., 2016). In addition to their role at the central level, NKB and NK3R are involved in the regulation of peripheral reproductive functions. Both NKB and NK3R are present in ovary, testes, and prostate (Cejudo Roman et al., 2012). NKB is also expressed in the outer syncytiotrophoblast of the placenta, and its overexpression has been also associated with pregnancy-induced hypertension and preeclampsia (Cejudo Roman et al., 2012).

Real-time PCR analysis, performed on AGT CRH and TAC3 genes confirmed the trend of microarray modulation (Supplementary Figure S4).

## Discussion

**FIGURE 4:**
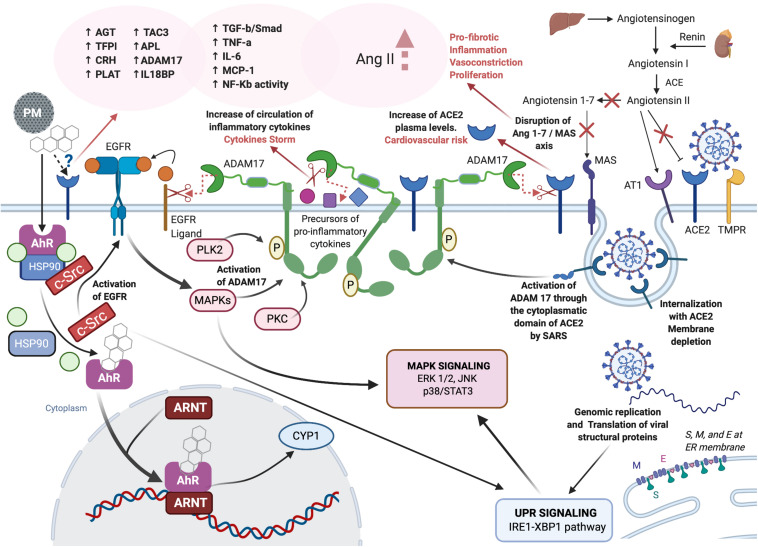
Role of PM-mediated inflammation in COVID-19. Created with Biorender.com.

Literature reports together with the new experimental data described in this paper, support the hypothesis that PM, directly or through molecular interplaying, affects the same molecular targets as so far known for SARS-CoV-2. The possible interplaying is depicted in Figure 4.

Even if the composition of PM is influenced by several factors, including geographical distribution, PM samples from Po valley and Wuhan city, the first and most severely affected areas globally, during the early months of COVID-19 outbreak, showed the same ability to induce the angiotensin II-dependent proinflammatory cytokine production in cell models (Colacci et al., 2014; Vaccari et al., 2015; Xu et al., 2016).

Modeling the exposure in cell models and using molecular signatures to highlight the mode and mechanism of action of single chemicals and complex mixtures allow the mechanistic interpretation of apical key events described in organs and related to adverse outcomes. This is particularly true for molecular key events related to innate immune response and immune-mediated inflammation (Jacobs et al., 2016, 2020; Mascolo et al., 2018).

The common behavior of chemical and biological xenobiotics underpins the agnostic nature of the main initiating event (MIE) in the pathway toward an adverse outcome. The adverse outcome pathway (AOP) is a paradigmatic representation of sequential steps starting with the MIE, which triggers the first response and orchestrates the following steps, and leading to the final outcome (OECD, 2018). AOP steps are marked by early key events, at the molecular, cellular and tissue levels, and by late steps related to apical endpoints, from organ toxicity to organ failures, to the full-blown disease in the individual, to the diffusion of the disease at the population level, highlighted by current epidemiological evidence. AOPs are considered independent (agnostic) from the agent of exposure, and are strictly related to the MIE, which means that whatever the agent hitting the MIE is, the final outcome might be the same, particularly if some early key events are shared.

It is becoming more and more evident that receptor binding and subsequent downstream DNA activation or deactivation is one of the most relevant MIE’s. It works both as the gateway for the xenobiotics entry into cellular homeostatic functions and as the trigger of the subsequent response to this entry. Furthermore, a single MIE could be the cause of multiple toxicological outcomes or a single apical endpoint may be the results of several MIEs. The interplay and cross talk amongst distinct receptors may be the key bridging several MIEs leading to a specific complex intercommunication network outcome.

It is noted however, that the essential physiological role of a receptor is maintaining the cell homeostasis, the cellular distribution of receptors varies according to tissue type and function, and its first role as MIE is ensuring the activation of immune-mediated defense and of detoxification pathways, and, eventually cell adaptation. In each AOP it can be possible to identify a committed step, which marks the turning point from adaptive to adverse response. The committed step is the result of the influence length of cellular exposure plus sufficient dose/concentration at the MIE to activate the next downstream events, the internal domains (from cellular to organ), genetic susceptibility and phenotype integrity, as well as of external domains, such as the temporal and quantitative exposure, and the concurrent action of multiple agents on the same MIEs.

Our hypothesis is based upon the possible interaction of PM and SARS-CoV-2 on the same MIE or on MIEs that interplay on the same critical outcomes, particularly endothelial disease, coagulation and metabolism disorders, and specifically genetic vulnerabilities that can predispose to these outcomes, such as familial history of diabetes.

We therefore discuss this possible interplay on the main adverse outcomes and risk factors that have been recognized for both PM exposure and COVID-19.

### PM, COVID-19 and Endothelial Disease

Besides respiratory disease, COVID-19 has been associated with ischemic complications, coagulation disorders and endotheliitis.

All these complications are due to the disruption of the endothelium homeostasis.

It is well known that endothelial dysfunction precedes the development of atherosclerosis, increasing the cardiovascular risk. Endothelial dysfunction is associated with most forms of cardiovascular disease, such as hypertension, CAD, chronic heart failure, peripheral artery disease, but also to diabetes and chronic renal failure. The up-regulation of adhesion molecules, increased chemokine secretion and leukocyte adherence have all been described as early key events in endothelial dysfunction, followed by increased cell permeability, platelet activation cytokine elaboration, and VSMC proliferation and migration (Endemann and Schiffrin, 2004; Hadi et al., 2005).

As the endothelial dysfunction was first reported in 1990 in human hypertension and was initially associated with the vasodilation induced by acetylcholine and bradykinin, the role of RAS on the endothelium homeostasis becomes clear.

The disrupting activity of SARS-CoV-2 towards ACE2 is one, and maybe the most important, cause for endothelial dysfunction.

ACE2 is expressed in endothelial cells of lung and other organs (Teuwen et al., 2020; Varga et al., 2020). Reduced ACE2 activity indirectly activates the kallikrein–bradykinin pathway, increasing vascular permeability (Pober and Sessa, 2007; Teuwen et al., 2020).

However, the direct effects of SARS-CoV-2 on endothelium has also been seen clinically in patients suffering severe symptoms of the disease, showing direct virus infiltration and infection of endothelial cells, as well as diffuse endothelial inflammation (Varga et al., 2020).

We have previously postulated the role of PM in endothelial disease, based on the molecular pathways obtained in other *in vitro* models (Colacci et al., 2014). Adenocarcinomic human alveolar basal epithelial (A549) cells, exposed to PM, showed the modulation of gene pathways involved in leukocyte TEM, with the upregulation of cell adhesion molecules (CAMs) active in vasculopathy and vasculitis, as complications of the autoimmune systemic lupus erythematosus disease, which is characterized by the deposition of immune complexes in endothelium, endothelial activation and inflammatory cell infiltration (Colacci et al., 2014).

The results following the exposure of T47D to PM winter samples gave evidence for the modulation of gene pathways involved in cell adhesion and TEM, including the up- or down-regulation of genes involved in the regulation of cytoskeleton and cell adhesion. Adhesion molecules are particularly implicated in a wide variety of cardiovascular disorders that involve inflammation, such as atherogenic processes and progression of atherosclerotic plaque, myocardial infarction, ischemia-reperfusion injury or transplant rejection, and, to a lesser extent, valve stenosis, and myocardiopathy (Barreiro and Sánchez-Madrid, 2009).

PM may directly affect the kallikrein-bradykinin pathway by disrupting the ACE axis, accelerating the process of vasodilation.

The whole picture of the molecular signatures supports the hypothesis that PM can affect pathways that are also involved in the endothelial dysfunctions observed in COVID-19 + patients.

### PM, COVID-19 and Coagulation Disorders

It is known that angiotensin-II-dependent hypertension is associated with microvascular (arterioles) thrombosis. Indeed, the risk of thrombosis in hypertensive patients decreases by treatments with ACE inhibitors or angiotensin-II receptor blockers (ARBs). Coagulation abnormalities and accelerated thrombosis caused by angiotensin II are marked by the activation of a plethora of receptors, including AT1, AT2, AT4 receptors, bradykinin 1 and 2, and endothelin-1 receptors. All these receptors are implicated in angiotensin II-mediated arteriolar thrombosis (Senchenkova et al., 2015).

Many patients affected by severe COVID-19 present coagulation abnormalities and are at risk of developing disseminated intravascular coagulation (DIC). While coagulation abnormalities have been described in other diseases, those associated with COVID-19 show peculiar features. In the first few weeks of COVID-19 outbreaks, these coagulation abnormalities were observed only post-mortem, as microvascular platelet-rich thrombotic depositions in small vessels of the lungs and other organs, which, despite markedly elevated levels of d-dimers, were not associated with the depletion of circulating platelets, as usually observed in thrombotic microangiopathy.

As the knowledge of the complexity of COVID-19 has improved, the blood coagulation changes in COVID + patients were increasingly regarded as markers of poor prognosis and of increased risk of death, since observing the dramatic effects that the combination of low grade DIC with the peculiar localized pulmonary thrombotic microangiopathy can have, on organ dysfunction and failure (Levi et al., 2020). This shifted the attention from the primary pathogenesis described as the acute respiratory distress syndrome to the so-called “pulmonary intravascular coagulopathy” (PIC), associated with the immune-response and considered an immune-mediated fibrosis (Belen-Apak and Sarıalioǧlu, 2020; McGonagle et al., 2020).

We have previously reported that the *in vitro* exposure of fibroblasts to PM samples induced the up-regulation of key-genes associated with idiopathic pulmonary fibrosis (Vaccari et al., 2015). The analysis of the molecular changes induced in T47D cells exposed to PM samples reveals that the most affected pathways are those related to coagulation and the plasminogen cascade. Some evidence of the effect of the exposure to PM on coagulation parameters and on the blood levels of molecules involved in the cascade of coagulation has been reported (Bonzini et al., 2010). However, despite the well-known role of PM in cardiovascular disease, the correlation between PM and coagulation changes in humans remains largely unexplored.

As we stated before, T47D cells express both AT1 and AT2 receptors. The overall picture of the molecular changes and modulated pathways following the exposure of these cells to PM samples is coherent with the activation of the cascade orchestrated by ACE. Due to the enigmatic nature of AT2 receptor, the structure of which has not been revealed yet, it is AT1R that is considered to be the master key in the initiation of the pathway leading to the adverse effects sustained by angiotensin II. Therefore, the results displayed here appear to confirm that PM can affect the coagulation through an AT1R-mediated pathway. However, the upregulation of apelin in cells exposed to PM, is suggestive of the direct involvement of ACE2. We can, therefore, speculate that PM affects the coagulation cascade by the same mechanism postulated for SARS-CoV-2 in COVID + patients.

### PM, COVID-19, and Diabetes

The role of RAS in diabetes and metabolic syndrome was first described in the ‘70s and has become clear during the last 30 years. Diabetes mellitus is often associated with hypertension, retinopathy, nephropathy and cardiovascular disease, which are initiated and sustained by the disruption of RAS. The implication of RAS was confirmed by the successful therapeutic use of RAS inhibitors and RAS receptor blockers in inhibiting the onset of diabetes complications, and also enhancing insulin sensitivity associated with decreased adipocyte size and increasing transcapillary glucose transport (Ribeiro-Oliveira et al., 2008). Therefore, RAS can play a direct role in diabetes due to both its endocrine and paracrine and intracrine activity at the tissue level. The degradation pathway of angiotensin II is considered the key step in the control of diabetes and its complications. Angiotensin II can cause insulin resistance by interfering with the insulin-stimulated increase in insulin receptor substrate 1-associated PI3K activity. Its degradation into smaller peptides, namely angiotensin III, which is further metabolized to angiotensin IV, by the enzymatic activity of aminopeptidase A (APA), activates the AT4 receptor, which has been identified as the transmembrane enzyme, insulin-regulated membrane aminopeptidase (IRAP). IRAP is a type II integral membrane spanning protein belonging to the M1 family of aminopeptidases and is predominantly found in GLUT4 vesicles in insulin-responsive cells. For this reason, the non-classical RAS ANG IV/AT4/IRAP axis has been suggested to modulate glucose uptake by modulating trafficking of GLUT4 (Li et al., 2017).

PM2.5 is associated with increased risk of diabetes (Bowe et al., 2018). PM2.5 exposure has been found to aggravate type 2 diabetes, by enhancing insulin resistance through the activation of the classical cascade of inflammatory cytokines, including IL-6 TNF-α, and MCP-1, in lung as a key event at organ level, and IL-6-dependent activation of STAT3/SOC3 pathway in liver as a key event at organism level, leading to a systemic effect (Long et al., 2020). These effects are likely to be mediated at least in part, by the AhR, whose role in hyperglycemia and vascular complications in diabetic patients, has been described and ascribed to its ability to form a complex with several transcription factors activated by glucose, including thrombospondin 1, a protein implicated in the development of vascular complications in diabetic patients (Dabir et al., 2008).

Our experimental data confirm that the exposure to PM2.5 can elicit the activation of gene pathways related to diabetes mellitus with the modulation of GAD1 as a consequence of the disruption of angiotensin II/AT1 axis, strongly suggesting an interplay between the AhR pathway, as the environmental sensor, and RAS.

Diabetes has been reported as a risk for patients COVID+. Patients with diabetes have higher levels of proinflammatory cytokines, more severe respiratory symptoms and worse survival rate (Shenoy et al., 2020). However, a few literature reports give evidence for a role of both SARS-CoV and SARS-CoV2 in the development of diabetes in healthy patients. Coronavirus is able to induce both type-1 and type-2 diabetes. Most of the cases observed in SARS patients resolved after a few years. However, diabetes persisted in 10% of patients (Yang et al., 2010). It has been proposed that coronavirus penetrate into pancreatic islet cells expressing high levels of ACE2, disrupting cell function and impairing insulin secretion (Yang et al., 2010).

### PM, COVID-19 and Reproductive Adverse Outcomes

One of the most challenging results derived from our study, was the modulation of the “female pregnancy” pathway and the upregulation of genes involved in pre-term births (PTBs).

This experimental result provides the mechanistic interpretation of epidemiological evidence in the resident population in the same area where the PM samples had been collected (Candela et al., 2013) and confirmed in a more recent publication (Ottone et al., 2020).

The rate of PTBs, i.e., delivering at less than 37 weeks, is rising globally. Air pollution, and especially PM, is thought to be responsible for nearly 3 million premature births (PTB), which means 18% of all annual PTBs (Murray and Lopez, 1997; GBD, 2016).

The developmental role and high expression of the AhR in human placenta may explain the relative easy entry of agents that can affect birth delivery. The activation of the AhR pathway could be the MIE of the AOP leading to PTB.

Prematurity is not the only adverse effect that has been linked to environmental pollution. Spontaneous abortion, intrauterine growth retardation, and low birth weight are all adverse pregnancy outcomes that are reported as the possible consequence of exposure to disrupting environmental agents (Candela et al., 2015). PTBs, however, are the leading cause of neonatal morbidity and mortality. Surviving babies are at increased risk of neurodevelopmental impairments, respiratory and gastrointestinal complications, and more inclined to develop diseases in adult life.

Previous reports on the effect of the outbreak of SARS in 2002–2003 on pregnancy, have shown that the infection by SARS-CoV during the perinatal period was associated with several adverse reproductive outcomes, including CID, abortion, preterm birth and intrauterine growth retardation. However, the data are insufficient to draw any conclusion on the effect of COVID-19 on pregnancy outcomes and even on the possible vertical transmission of the infection from mother to fetus. Interestingly, some anecdotal reports and the initial literature reports are beginning to suggest that there might be evidence for an increased risk for preterm birth in mothers that are COVID+ (Panahi et al., 2020; Qiao, 2020; Rasmussen et al., 2020).

Of course a key cofounder of this evidence will be the influence of the emotional stress on pregnancy adverse outcomes, but even so, the pivotal role of angiotensinogen in the adverse pathway to PTB, as revealed in our experiments, is suggestive of a common key player that can be activated by chemical and biological agents sharing the same MIEs.

## Conclusion and Future Perspectives

The scientific reports and experimental data discussed herein suggest a role of PM in exacerbating the inflammation induced by COVID-19, on the basis of plausible interplay at the molecular level.

In which case, the additive exposure to PM could be regarded as another risk factor for developing severe forms of COVID-19, together with male gender, age over 65 and smoking habit. Smoking and PM exposure, as complex mixtures, share some of the same chemical components that could be responsible for the activation of the AhR–RAS-ACE2 crosstalk network. Smoking probably contributes to the creation of a hypoxic microenvironment that can contribute to the dysregulation of RAS.

However, even if the putative role of PM in severe and mortal forms of COVID-19 is intriguing and deserving of further investigations, other contributory factors need to be considered in an appropriately designed epidemiological study to establish whether there is an association between air pollution and COVID-19. It is further noted that whilst the T47D cell line provides a suitable test method, reproduction of these results utilizing lung and alveolar cell lines, and primary cell cultures is needed. This experimental work is already underway, to continue to tease out the causality links.

Based on the data presented in this paper, exposure to high concentrations of PM could contribute to the cytokine storm and to the aggravation of the symptoms triggered by COVID-19, rather than sustaining the spread of the infection by facilitating the entry of the virus. On the basis of this discussion, we have two possibilities to consider. The first possibility is that the internalization of PM components and of the virus operate through two different receptors, AhR and ACE2, respectively. In this case the combination of the effects is the result of an intense molecular interplay and disruption of the same molecular targets, and the downstream signaling pathways that are orchestrated by the two receptors. The second possibility is that ACE 2 serves as the receptor for PM components. In that case, PM components and viral particles would compete for the same receptor and be hitting the same signaling pathways. Even if a direct link of PM with ACE2 has not been demonstrated yet, the second possibility cannot be ruled out.

The central role of ACE2 in regulating the cell and tissue homeostasis, counterbalancing the effects sustained by RAS, is reinforced by the analysis of its involvement in COVID-19 and by the molecular changes in response to PM exposure. Since the outbreak of SARS in 2003, ACE2 has been in the spotlight. The evidence of its involvement as the receptor of at least three distinct coronaviruses stimulate some questions as to its role in the virus evolution as well as in the fetal development and childhood. Children appear to be more resistant to SARS-CoV-2 infection and less prone to develop symptoms of the disease. Is the higher expression of AT2 receptor, which is regulated by ACE2, during the fetal life and childhood a possible explanation for the lack of maternal transmission during pregnancy and higher resistance to the infection in childhood? And is the attenuation of this expression responsible for the peripheral vasculitis and Kawasaki-like syndromes that have been anecdotally reported in children that are COVID+? (Safadi and Silva, 2020). Interestingly, ACE2 seems to play a role in each of the risk factors that have been associated with COVID-19. Besides the protective role of ACE2 towards all co-morbidities for COVID-19, such as hypertension, diabetes, cardiovascular disease, and respiratory diseases, ACE2 plays also a protective role against the aging processes supported by RAS. Moreover, the ACE2 gene is located on chromosome X, not distant from genes coding for toll-like receptors 7 and 8 (TLR7 and TLR8), which functionally recognize single strand RNA viruses, and have been discussed as potentially responsible for the gender-specific response that have been observed in both SARS-CoV and SARS-CoV2 outbreaks. Finally, ACE2, as well as TMPRSS2 polymorphisms may be responsible for the different susceptibility to infection.

Learning these valuable lessons from the COVID-19 pandemic, clarification on the use of RAS molecules and ACE2 as markers of prognosis in patients that are COVID+ would be of great assistance, and also more broadly, for all the adverse outcomes where they play a role. Further investigations into the role of PM as a booster in the inflammation response sustained by the disruption of receptor-mediated signaling pathways would ensure helpful new insights into the mode and mechanism of action of environmental exposures and their possible role in exacerbating all the respiratory diseases, including seasonal flu and colds, all of which are sustained by viruses with pandemic potential.

Finally, the data discussed in this paper offer new insights into the molecular interplay, the secretive liaison of chemicals and pathogens together, that could be of importance for sustaining public health policies and developing new therapeutic approaches.

## Dedication

This manuscript is dedicated to the memory of Professor Emeritus Sandro Grilli, mentor, colleague and friend, who passed away on 13th May 2020, as a victim of an unknown virus. He was a pathologist and an oncologist, an expert of cancer risk assessment and of environmental toxicology. His dedication to research and teaching, his commitment to his family and friends, his loyalty to colleagues, his kindness and generosity are a source of inspiration for all of us and for all whose life he touched. He will truly be missed.

## Data Availability Statement

Microarray experimental design and protocols, together with the complete raw data-set, are available in the EBI microarray data public repository Arrayexpress http://www.ebi.ac.uk/arrayexpress/, accession number E-MEXP-3686.

## Author Contributions

AM and GM conceived and drafted the original hypothesis of the molecular interplay. MM, EM, and FR carried out the experiments. MM and GP processed the experimental data, performed the analysis, drafted the results, and designed the figures with the support of AM, GM, and SS. GB supervised the project and encouraged AC to investigate. AC contributed to the design and implementation of the research, to the analysis of the results, and wrote the manuscript with inputs from AR, GB, MJ, SM, and SZ and in consultation with MM, AM, GM, GP, and SS. MJ contributed further to the final version and critical revision of the manuscript. All the authors provided critical feedback and helped shape the research, analysis and manuscript.

## Disclaimer

The view expressed in this article are those of the authors, and do not necessarily represent the views of Public Health England.

## Conflict of Interest

The authors declare that the research was conducted in the absence of any commercial or financial relationships that could be construed as a potential conflict of interest.

## References

[B1] Aztatzi-Aguilar O. G. Uribe-Ramírez M. Arias-Montaño J. A. Barbier O. De Vizcaya-Ruiz A. (2015). Acute and subchronic exposure to air particulate matter induces expression of angiotensin and bradykinin-related genes in the lungs and heart: angiotensin-II type-I receptor as a molecular target of particulate matter exposure. *Part. Fibre Toxicol.* 12:17. 10.1186/s12989-015-0094-4 26113123 PMC4482198

[B2] Baldini M. Bartolacci S. Bortone G. Colacci A. Di Biagio K. Di Buono V. (2020). Valutazione del possibile rapporto tra l’inquinamento atmosferico e la diffusione del SARS-CoV-2 - E&P repository. *Epidemiol. Prev.* [Epub ahead of print].

[B3] Barreiro O. Sánchez-Madrid F. (2009). Molecular basis of leukocyte–endothelium interactions during the inflammatory response. *Rev. Española Cardiol.* 62 552–562. 10.1016/s1885-5857(09)71837-7 19406069

[B4] Belen-Apak F. B. Sarıalioǧlu F. (2020). Pulmonary intravascular coagulation in COVID-19: possible pathogenesis and recommendations on anticoagulant/thrombolytic therapy. *J. Thromb. Thrombolysis* [Epub ahead of print]. 10.1007/s11239-020-02129-0 32372336 PMC7200048

[B5] Bonzini M. Tripodi A. Artoni A. Tarantini L. Marinelli B. Bertazzi P. A. (2010). Effects of inhalable particulate matter on blood coagulation. *J. Thromb. Haemost.* 8 662–668. 10.1111/j.1538-7836.2009.03694.x 19922434 PMC3093960

[B6] Bourouiba L. (2020). Turbulent gas clouds and respiratory pathogen emissions: potential implications for reducing transmission of COVID-19. *J. Am. Med. Assoc.* 323 1837–1838. 10.1001/jama.2020.4756 32215590

[B7] Bowe B. Xie Y. Li T. Yan Y. Xian H. Al-Aly Z. (2018). The 2016 global and national burden of diabetes mellitus attributable to PM 25 air pollution. *Lancet Planet. Heal.* 2 e301–e312. 10.1016/S2542-5196(18)30140-2 30074893

[B8] Bujak-Gizycka B. Madej J. Bystrowska B. Toton-Zuranska J. Kus K. Kolton-Wroz M. (2019). Angiotensin 1-7 formation in breast tissue is attenuated in breast cancer-a study on the metabolism of angiotensinogen in breast cancer cell lines. *J. Physiol. Pharmacol.* 70 503–514. 10.26402/jpp.2019.4.02 31642813

[B9] Cai Y. Schikowski T. Adam M. Buschka A. Carsin A. E. Jacquemin B. (2014). Cross-sectional associations between air pollution and chronic bronchitis: an ESCAPE meta-analysis across five cohorts. *Thorax* 69 1005–1014. 10.1136/thoraxjnl-2013-204352 25112730

[B10] Candela S. Bonvicini L. Ranzi A. Baldacchini F. Broccoli S. Cordioli M. (2015). Exposure to emissions from municipal solid waste incinerators and miscarriages: a multisite study of the MONITER Project. *Environ. Int.* 78 51–60. 10.1016/j.envint.2014.12.008 25765761

[B11] Candela S. Ranzi A. Bonvicini L. Baldacchini F. Marzaroli P. Evangelista A. (2013). Air pollution from incinerators and reproductive outcomes. *Epidemiology* 24 863–870. 10.1097/EDE.0b013e3182a712f1 24076993

[B12] Cejudo Roman A. Pinto F. M. Dorta I. Almeida T. A. Hernández M. Illanes M. (2012). Analysis of the expression of neurokinin B, kisspeptin, and their cognate receptors NK3R and KISS1R in the human female genital tract. *Fertil. Steril.* 97 1213–1219. 10.1016/j.fertnstert.2012.02.021 22424618

[B13] Chan S. W. (2014). The unfolded protein response in virus infections. *Front. Microbiol.* 5:518. 10.3389/fmicb.2014.00518 25324837 PMC4179733

[B14] Colacci A. Vaccari M. (2017). “Children’s and adult involuntary and occupational exposures and cancer,” in *Translational Toxicology and Therapeutics: Windows of Developmental Susceptibility in Reproduction and Cancer*, eds Waters M. D. Hughes C., (Hoboken: John Wiley & Sons, Inc), 259–316.

[B15] Colacci A. Vaccari M. Mascolo M. G. Rotondo F. Morandi E. Quercioli D. (2014). Alternative testing methods for predicting health risk from environmental exposures. *Sustainability* 6 5265–5283. 10.3390/su6085265

[B16] COMEAP (2018). *Committee on the Medical Effects of Air Pollutants Reports and Statements Air pollution and Cardiovascular Disease: Mechanistic Evidence.* London: Public Health England Available online at: https://www.gov.uk/government/collections/comeap-reports

[B17] Conticini E. Frediani B. Caro D. (2020). Can atmospheric pollution be considered a co-factor in extremely high level of SARS-CoV-2 lethality in Northern Italy? *Environ. Pollut.* 261:114465. 10.1016/j.envpol.2020.114465 32268945 PMC7128509

[B18] Contini D. Costabile F. (2020). Does air pollution influence COVID-19 outbreaks? *Atmosphere* 11:377 10.3390/ATMOS11040377

[B19] Coutard B. Valle C. de Lamballerie X. Canard B. Seidah N. G. Decroly E. (2020). The spike glycoprotein of the new coronavirus 2019-nCoV contains a furin-like cleavage site absent in CoV of the same clade. *Antiviral Res.* 176:104742. 10.1016/j.antiviral.2020.104742 32057769 PMC7114094

[B20] Dabir P. Marinic T. E. Krukovets I. Stenina O. I. (2008). Aryl Hydrocarbon receptor is activated by glucose and regulates the Thrombospondin-1 gene promoter in endothelial cells. *Circ. Res.* 102 1558–1565. 10.1161/CIRCRESAHA.108.176990 18515748 PMC2740473

[B21] Darrow L. A. Klein M. Dana Flanders W. Mulholland J. A. Tolbert P. E. Strickland M. J. (2014). Air pollution and acute respiratory infections among children 0-4 years of age: an 18-year time-series study. *Am. J. Epidemiol.* 180 968–977. 10.1093/aje/kwu234 25324558 PMC4224364

[B22] DeDiego M. L. Nieto-Torres J. L. Jiménez-Guardeño J. M. Regla-Nava J. A. Álvarez E. Oliveros J. C. (2011). Severe acute respiratory syndrome coronavirus envelope protein regulates cell stress response and apoptosis. *PLoS Pathog.* 7:e1002315. 10.1371/journal.ppat.1002315 22028656 PMC3197621

[B23] Delforce S. J. Lumbers E. R. Ellery S. J. Murthi P. Pringle K. G. (2019). Dysregulation of the placental renin–angiotensin system in human fetal growth restriction. *Reproduction* 158 237–245. 10.1530/REP-18-0633 31247590

[B24] Dolan J. M. Sim F. J. Meng H. Kolega J. (2012). Endothelial cells express a unique transcriptional profile under very high wall shear stress known to induce expansive arterial remodeling. *Am. J. Physiol. Cell Physiol.* 302 C1109–C1118. 10.1152/ajpcell.00369.2011 22173868 PMC3330730

[B25] Egan K. O’Connor H. Kevane B. Malone F. Lennon A. Al Zadjali A. (2017). Elevated plasma TFPI activity causes attenuated Tf-Dependent thrombin generation in early onset preeclampsia. *Thromb. Haemost.* 117 1549–1557. 10.1160/TH16-12-0949 28569919

[B26] Endemann D. H. Schiffrin E. L. (2004). Endothelial dysfunction. *J. Am. Soc. Nephrol.* 15 1983–1992. 10.1097/01.ASN.0000132474.50966.DA 15284284

[B27] Fung T. S. Liu D. X. (2014). Coronavirus infection, ER stress, apoptosis and innate immunity. *Front. Microbiol.* 5:296. 10.3389/fmicb.2014.00296 24987391 PMC4060729

[B28] Gauderman W. J. Urman R. Avol E. Berhane K. McConnell R. Rappaport E. (2015). Association of improved air quality with lung development in children. *N. Engl. J. Med.* 372 905–913. 10.1056/NEJMoa1414123 25738666 PMC4430551

[B29] GBD (2016). *Global Burden of Disease Study 2016 Data Resources | GHDx.* Washington, DC: GBD.

[B30] Ghio A. J. Carraway M. S. Madden M. C. (2012). Composition of air pollution particles and oxidative stress in cells, tissues, and living systems. *J. Toxicol. Environ. Heal. B Crit. Rev.* 15 1–21. 10.1080/10937404.2012.632359 22202227

[B31] Glise L. Larsson P. Jern S. Borén J. Levin M. Ny T. (2019). Disturbed laminar blood flow causes impaired fibrinolysis and endothelial fibrin deposition in vivo. *Thromb. Haemost.* 119 223–233. 10.1055/s-0038-1676638 30602198

[B32] Glotov A. S. Tiys E. S. Vashukova E. S. Pakin V. S. Demenkov P. S. Saik O. V. (2015). Molecular association of pathogenetic contributors to pre-eclampsia (pre-eclampsia associome). *BMC Syst. Biol.* 9:S4. 10.1186/1752-0509-9-S2-S4 25879409 PMC4407242

[B33] Gui G. P. H. Puddefoot J. R. Vinson G. P. Wells C. A. Carpenter R. (1997). Altered cell-matrix contact: a prerequisite for breast cancer metastasis? *Br. J. Cancer* 75 623–633. 10.1038/bjc.1997.113 9043016 PMC2063333

[B34] Hadi H. A. R. Carr C. S. Al Suwaidi J. (2005). Endothelial dysfunction: cardiovascular risk factors, therapy, and outcome. *Vasc. Health Risk Manag.* 1 183–198. 17319104 PMC1993955

[B35] Hamming I. Cooper M. Haagmans B. Hooper N. Korstanje R. Osterhaus A. (2008). The emerging role of ACE2 in physiology and disease. *J. Pathol.* 212 1–11. 10.1002/path.2162 17464936 PMC7167724

[B36] Hamra G. B. Guha N. Cohen A. Laden F. Raaschou-Nielsen O. Samet J. M. (2014). Outdoor particulate matter exposure and lung cancer: a systematic review and meta-analysis. *Environ. Health Perspect.* 122 906–911. 10.1289/ehp.1408092 24911630 PMC4154221

[B37] Harrison R. M. Yin J. (2000). Particulate matter in the atmosphere: which particle properties are important for its effects on health? *Sci. Total Environ.* 249 85–101. 10.1016/S0048-9697(99)00513-6 10813449

[B38] Heidari M. Razaviyan Z. Yusof F. Mohammadian E. Alias A. Akhbari M. (2019). Numerical analysis of side hull configuration in Trimaran. *Rev. Int. Métodos Numéricos para Cálculo y Diseño en Ing.* 35:32 10.23967/j.rimni.2019.06.004

[B39] Hoffmann M. Kleine-Weber H. Schroeder S. Krüger N. Herrler T. Erichsen S. (2020). SARS-CoV-2 cell entry depends on ACE2 and TMPRSS2 and is blocked by a clinically proven protease inhibitor. *Cell* 181 271.e8–280.e8. 10.1016/j.cell.2020.02.052 32142651 PMC7102627

[B40] Ichihara S. Li P. Mise N. Suzuki Y. Izuoka K. Nakajima T. (2019). Ablation of aryl hydrocarbon receptor promotes angiotensin II-induced cardiac fibrosis through enhanced c-Jun/HIF-1α signaling. *Arch. Toxicol.* 93 1543–1553. 10.1007/s00204-019-02446-1 31016362 PMC7395242

[B41] Inwang E. R. Puddefoot J. R. Brown C. L. Goode A. W. Marsigliante S. Ho M. M. (1997). Angiotensin II type 1 receptor expression in human breast tissues. *Br. J. Cancer* 75 1279–1283. 10.1038/bjc.1997.217 9155046 PMC2228240

[B42] Jacobs M. N. (2005). “Nuclear receptor and dietary ligands,” in *Nutrients and Cell Signaling*, eds Zemplini J. Dakshinamurti K., (New York, NY: CRC Press), 35–89.

[B43] Jacobs M. N. Colacci A. Corvi R. Vaccari M. Aguila M. C. Corvaro M. (2020). Chemical carcinogen safety testing: OECD expert group international consensus on the development of an integrated approach for the testing and assessment of chemical non-genotoxic carcinogens. *Arch. Toxicol.* 94 2899–2923. 10.1007/s00204-020-02784-5 32594184 PMC7395040

[B44] Jacobs M. N. Colacci A. Louekari K. Luijten M. Hakkert B. C. Paparella M. (2016). International regulatory needs for development of an IATA for non-genotoxic carcinogenic chemical substances. *ALTEX* 33 359–392. 10.14573/altex.1601201 27120445

[B45] Jacquemin B. Siroux V. Sanchez M. Carsin A. E. Schikowski T. Adam M. (2015). Ambient air pollution and adult asthma incidence in six european cohorts (Escape). *Environ. Health Perspect.* 123 613–621. 10.1289/ehp.1408206 25712593 PMC4455584

[B46] Jia H. P. Look D. C. Shi L. Hickey M. Pewe L. Netland J. (2005). ACE2 receptor expression and severe acute respiratory syndrome coronavirus infection depend on differentiation of human airway epithelia. *J. Virol.* 79 14614–14621. 10.1128/jvi.79.23.14614-14621.2005 16282461 PMC1287568

[B47] Kilpatrick L. E. Kiani M. F. (2020). Experimental approaches to evaluate leukocyte–endothelial cell interactions in sepsis and inflammation. *Shock* 53 585–595. 10.1097/SHK.0000000000001407 32080065 PMC7160007

[B48] Levi M. Thachil J. Iba T. Levy J. H. (2020). Coagulation abnormalities and thrombosis in patients with COVID-19. *Lancet Haematol.* 7 e438–e440. 10.1016/S2352-3026(20)30145-9 32407672 PMC7213964

[B49] Li X. C. Zhang J. Zhuo J. L. (2017). The vasoprotective axes of the renin-angiotensin system: physiological relevance and therapeutic implications in cardiovascular, hypertensive and kidney diseases. *Pharmacol. Res.* 125 21–38. 10.1016/j.phrs.2017.06.005 28619367 PMC5607101

[B50] Lin C. I. Tsai C. H. Sun Y. L. Hsieh W. Y. Lin Y. C. Chen C. Y. (2018). Instillation of particulate matter 2.5 induced acute lung injury and attenuated the injury recovery in ACE2 knockout mice. *Int. J. Biol. Sci.* 14 253–265. 10.7150/ijbs.23489 29559844 PMC5859472

[B51] Long M. Zhang C. Xu D. Fu W. Gan X. Li F. (2020). PM2.5 aggravates diabetes via the systemically activated IL-6-mediated STAT3/SOCS3 pathway in rats’ liver. *Environ. Pollut.* 256:113342. 10.1016/j.envpol.2019.113342 31676093

[B52] Manisalidis I. Stavropoulou E. Stavropoulos A. Bezirtzoglou E. (2020). Environmental and health impacts of air pollution: a review. *Front. Public Heal.* 8:14. 10.3389/fpubh.2020.00014 32154200 PMC7044178

[B53] Mascolo M. G. Perdichizzi S. Vaccari M. Rotondo F. Zanzi C. Grilli S. (2018). The transformics assay: first steps for the development of an integrated approach to investigate the malignant cell transformation in vitro. *Carcinogenesis* 39 955–967. 10.1093/carcin/bgy037 29554273 PMC6031005

[B54] Masgrau-Alsina S. Sperandio M. Rohwedder I. (2020). Neutrophil recruitment and intracellular vesicle transport: a short overview. *Eur. J. Clin. Invest.* 50:e13237. 10.1111/eci.13237 32289185

[B55] Maunders H. Patwardhan S. Phillips J. Clack A. Richter A. (2007). Human bronchial epithelial cell transcriptome: gene expression changes following acute exposure to whole cigarette smoke in vitro. *Am. J. Physiol. Lung Cell. Mol. Physiol.* 292 1248–1256. 10.1152/ajplung.00290.2006 17220372

[B56] McGonagle D. O’Donnell J. S. Sharif K. Emery P. Bridgewood C. (2020). Immune mechanisms of pulmonary intravascular coagulopathy in COVID-19 pneumonia. *Lancet Rheumatol.* 2 E437–E445. 10.1016/S2665-9913(20)30121-1 32835247 PMC7252093

[B57] Menzies F. M. Oldham R. S. Waddell C. Nelson S. M. Nibbs R. J. B. (2020). A comprehensive profile of chemokine gene expression in the tissues of the female reproductive tract in mice. *Immunol. Invest.* 49 264–286. 10.1080/08820139.2019.1655573 31429329

[B58] Miyashita L. Foley G. Semple S. Grigg J. (2020). Traffic-derived particulate matter and angiotensin-converting enzyme 2 expression in human airway epithelial cells. *BioRxiv* [Preprint]. 10.1101/2020.05.15.097501

[B59] Mulero-Navarro S. Fernandez-Salguero P. M. (2016). New trends in Aryl hydrocarbon receptor biology. *Front. Cell Dev. Biol.* 4:45. 10.3389/fcell.2016.00045 27243009 PMC4863130

[B60] Murray C. J. L. Lopez A. D. (1997). Global mortality, disability, and the contribution of risk factors: global burden of disease study. *Lancet* 349 1436–1442. 10.1016/S0140-6736(96)07495-8 9164317

[B61] Naji D. H. Tan C. Han F. Zhao Y. Wang J. Wang D. (2018). Significant genetic association of a functional TFPI variant with circulating fibrinogen levels and coronary artery disease. *Mol. Genet. Genomics* 293 119–128. 10.1007/s00438-017-1365-6 28894953 PMC5794607

[B62] Ng H.-Y. Bolati W. Lee C.-T. Chien Y.-S. Yisireyili M. Saito S. (2016). Indoxyl sulfate downregulates mas receptor via aryl hydrocarbon receptor/nuclear factor-Kappa B, and induces cell proliferation and tissue factor expression in vascular smooth muscle cells. *Nephron* 133 205–212. 10.1159/000447096 27352232

[B63] OECD (2018). *OECD Series on Adverse Outcome Pathways No. 1 Users’ Handbook supplement to the Guidance Document for developing and assessing Adverse Outcome Pathways.* Paris: OECD.

[B64] Ottone M. Broccoli S. Parmagnani F. Giannini S. Scotto F. Bonvicini L. (2020). Source-related components of fine particulate matter and risk of adverse birth outcomes in Northern Italy. *Environ. Res.* 186:109564. 10.1016/j.envres.2020.109564 32668539

[B65] Oudit G. Y. Crackower M. A. Backx P. H. Penninger J. M. (2003). The role of ACE2 in cardiovascular physiology. *Trends Cardiovasc. Med.* 13 93–101. 10.1016/S1050-1738(02)00233-5 12691672

[B66] Panahi L. Amiri M. Pouy S. (2020). Risks of novel coronavirus disease (COVID-19) in pregnancy; a narrative review. *Arch. Acad. Emerg. Med.* 8:e34. 10.22037/aaem.v8i1.595 32232217 PMC7092922

[B67] Parienta D. Morawska L. Johnson G. R. Ristovski Z. D. Hargreaves M. Mengersen K. (2011). Theoretical analysis of the motion and evaporation of exhaled respiratory droplets of mixed composition. *J. Aerosol Sci.* 42 1–10. 10.1016/j.jaerosci.2010.10.005

[B68] Patel V. B. Mori J. McLean B. A. Basu R. Das S. K. Ramprasath T. (2016). ACE2 deficiency worsens epicardial adipose tissue inflammation and cardiac dysfunction in response to diet-induced obesity. *Diabetes* 65 85–95. 10.2337/db15-0399 26224885 PMC4686955

[B69] Peel J. L. Tolbert P. E. Klein M. Metzger K. B. Flanders W. D. Todd K. (2005). Ambient air pollution and respiratory emergency department visits. *Epidemiology* 16 164–174. 10.1097/01.ede.0000152905.42113.db 15703530

[B70] Pober J. S. Sessa W. C. (2007). Evolving functions of endothelial cells in inflammation. *Nat. Rev. Immunol.* 7 803–815. 10.1038/nri2171 17893694

[B71] Pope C. A. Bhatnagar A. McCracken J. P. Abplanalp W. Conklin D. J. O’Toole T. (2016). Exposure to Fine Particulate Air Pollution Is Associated with Endothelial Injury and Systemic Inflammation. *Circ. Res.* 119 1204–1214. 10.1161/CIRCRESAHA.116.309279 27780829 PMC5215745

[B72] Pope C. A. Ezzati M. Dockery D. W. (2009). Fine-particulate air pollution and life expectancy in the United States. *N. Engl. J. Med.* 360 376–386. 10.1056/NEJMsa0805646 19164188 PMC3382057

[B73] Procopciuc L. M. Nemeti G. Buzdugan E. Iancu M. Stamatian F. Caracostea G. (2019). Renin-angiotensin system gene variants and risk of early- and late-onset preeclampsia: a single center case-control study. *Pregnancy Hypertens.* 18 1–8. 10.1016/j.preghy.2019.08.006 31442828

[B74] Qi X. Salem M. Zhou W. Sato-Shimizu M. Ye G. Smitz J. (2016). Neurokinin B exerts direct effects on the ovary to stimulate estradiol production. *Endocrinology* 157 3355–3365. 10.1210/en.2016-1354 27580802

[B75] Qiao J. (2020). What are the risks of COVID-19 infection in pregnant women? *Lancet* 395 760–762. 10.1016/S0140-6736(20)30365-2 32151334 PMC7158939

[B76] Raftogianni A. Roth L. C. García-González D. Bus T. Kühne C. Monyer H. (2018). Deciphering the contributions of CRH receptors in the brain and pituitary to stress-induced inhibition of the reproductive axis. *Front. Mol. Neurosci.* 11:305. 10.3389/fnmol.2018.00305 30214395 PMC6125327

[B77] Ramhorst R. Calo G. Paparini D. Vota D. Hauk V. Gallino L. (2019). Control of the inflammatory response during pregnancy: potential role of VIP as a regulatory peptide. *Ann. N. Y. Acad. Sci.* 1437 15–21. 10.1111/nyas.13632 29740848

[B78] Ramos I. F. Guardino C. M. Mansolf M. Glynn L. M. Sandman C. A. Hobel C. J. (2019). Pregnancy anxiety predicts shorter gestation in Latina and non-Latina white women: the role of placental corticotrophin-releasing hormone. *Psychoneuroendocrinology* 99 166–173. 10.1016/j.psyneuen.2018.09.008 30245329 PMC6231951

[B79] Rasmussen S. A. Smulian J. C. Lednicky J. A. Wen T. S. Jamieson D. J. (2020). Coronavirus Disease 2019 (COVID-19) and pregnancy: what obstetricians need to know. *Am. J. Obstet. Gynecol.* 222 415–426. 10.1016/j.ajog.2020.02.017 32105680 PMC7093856

[B80] Ribeiro-Oliveira A. Nogueira A. I Pereira R. M. Vilas Boas W. W. Souza dos Santos R. A. Simões e Silva A. C. (2008). The renin-angiotensin system and diabetes: an update. *Vasc. Health Risk Manag.* 4 787–803. 10.2147/VHRM.S1905 19065996 PMC2597759

[B81] Safadi M. A. P. Silva C. A. A. (2020). The challenging and unpredictable spectrum of covid-19 in children and adolescents O espectro desafiador e imprevisível da COVID-19 em crianças e adolescentes. *Sci. Electron. Libr.* 10.1590/1984-0462/2020/38/2020192 [Epub ahead of print]. 32901700 PMC7477941

[B82] Sajadi M. M. Habibzadeh P. Vintzileos A. Shokouhi S. Miralles-Wilhelm F. Amoroso A. (2020). Temperature and latitude analysis to predict potential spread and seasonality for COVID-19. *SSRN Electron. J.* 10.2139/ssrn.3550308 [Epub ahead of print]. 32525550 PMC7290414

[B83] Sen B. Mahadevan B. DeMarini D. M. (2007). Transcriptional responses to complex mixtures-A review. *Mutat. Res. Rev. Mutat. Res.* 636 144–177. 10.1016/j.mrrev.2007.08.002 17888717

[B84] Senapati S. Wang F. Ord T. Coutifaris C. Feng R. Mainigi M. (2018). Superovulation alters the expression of endometrial genes critical to tissue remodeling and placentation. *J. Assist. Reprod. Genet.* 35 1799–1808. 10.1007/s10815-018-1244-z 29959621 PMC6150905

[B85] Senchenkova E. Seifert H. Granger D. N. (2015). Hypercoagulability and platelet abnormalities in inflammatory bowel disease. *Semin. Thromb. Hemost.* 41 582–589. 10.1055/s-0035-1556590 26270113

[B86] Serra S. Vaccari M. Mascolo M. G. Rotondo F. Zanzi C. Polacchini L. (2019). Hazard assessment of air pollutants: the transforming ability of complex pollutant mixtures in the Bhas 42 cell model. *ALTEX* 36 623–633. 10.14573/altex.1812173 31210278

[B87] Sharma K. Lee H. H. Gong D. S. Park S. H. Yi E. Schini-Kerth V. (2019). Fine air pollution particles induce endothelial senescence via redox-sensitive activation of local angiotensin system. *Environ. Pollut.* 252 317–329. 10.1016/j.envpol.2019.05.066 31158660

[B88] Shen L. Zhang T. Lu H. (2017). Overexpression of AGT promotes bronchopulmonary dysplasis via the JAK/STAT signal pathway. *Oncotarget* 8 96079–96088. 10.18632/oncotarget.21712 29221188 PMC5707082

[B89] Shenoy A. Ismaily M. Bajaj M. (2020). Diabetes and COVID-19: a global health challenge. *BMJ Open Diabetes Res. Care* 8:e001450. 10.1136/bmjdrc-2020-001450 32345580 PMC7222578

[B90] Shono M. Urushihara M. Suga K. Watanabe N. Saijo T. Nakagawa R. (2019). Enhanced angiotensinogen expression in neonates during kidney development. *Clin. Exp. Nephrol.* 23 537–543. 10.1007/s10157-018-1662-3 30353264

[B91] Stadtmauer D. J. Wagner G. P. (2020). The primacy of maternal innovations to the evolution of embryo implantation. *Integr. Comp. Biol.* 60 742–752. 10.1093/icb/icaa030 32525521 PMC7546962

[B92] Stamatelou F. Deligeoroglou E. Vrachnis N. Iliodromiti S. Iliodromiti Z. Sifakis S. (2013). Corticotropin-releasing hormone and progesterone plasma levels association with the onset and progression of labor - PubMed. *Clin. Exp. Obstet. Gynecol.* 40 568–571. 24597258

[B93] Sullivan D. P. Dalal P. J. Sacks D. B. Muller W. A. (2020). Calcium signaling regulates leukocyte transendothelial migration through the action of endothelial cell IQGAP1, calmodulin, and CaMKIIδ. *FASEB J.* 34:1 10.1096/fasebj.2020.34.s1.05149

[B94] Teuwen L. A. Geldhof V. Pasut A. Carmeliet P. (2020). COVID-19: the vasculature unleashed. *Nat. Rev. Immunol.* 20 389–391. 10.1038/s41577-020-0343-0 32439870 PMC7240244

[B95] Tisoncik J. R. Korth M. J. Simmons C. P. Farrar J. Martin T. R. Katze M. G. (2012). Into the eye of the cytokine storm. *Microbiol. Mol. Biol. Rev.* 76 16–32. 10.1128/mmbr.05015-11 22390970 PMC3294426

[B96] Torricelli M. Novembri R. Bloise E. De Bonis M. Challis J. R. Petraglia F. (2011). Changes in placental CRH, urocortins, and CRH-receptor mRNA expression associated with preterm delivery and chorioamnionitis. *J. Clin. Endocrinol. Metab.* 96 534–540. 10.1210/jc.2010-1740 21106714

[B97] Tseng C. C. Li C. S. (2005). Collection efficiencies of aerosol samplers for virus-containing aerosols. *J. Aerosol Sci.* 36 593–607. 10.1016/j.jaerosci.2004.12.004 32287372 PMC7118727

[B98] Vaccari M. Mascolo M. G. Rotondo F. Morandi E. Quercioli D. Perdichizzi S. (2015). Identification of pathway-based toxicity in the BALB/c 3T3 cell model. *Toxicol. Vitr.* 29 1240–1253. 10.1016/j.tiv.2014.10.002 25450744

[B99] Varga Z. Flammer A. J. Steiger P. Haberecker M. Andermatt R. Zinkernagel A. S. (2020). Endothelial cell infection and endotheliitis in COVID-19. *Lancet* 395 1417–1418. 10.1016/S0140-6736(20)30937-5 32325026 PMC7172722

[B100] Walls A. C. Park Y. J. Tortorici M. A. Wall A. McGuire A. T. Veesler D. (2020). Structure, function, and antigenicity of the SARS-CoV-2 spike glycoprotein. *Cell* 181 281.e6–292.e6. 10.1016/j.cell.2020.02.058 32155444 PMC7102599

[B101] Wang B. Ithier M. C. Parobchak N. Yadava S. M. Schulkin J. Rosen T. (2018). Vitamin D stimulates multiple microRNAs to inhibit CRH and other pro-labor genes in human placenta. *Endocr. Connect.* 7 1380–1388. 10.1530/EC-18-0345 30395535 PMC6280586

[B102] Wang W. Han X. Zhao T. Zhang X. Qu P. Zhao H. (2020). AGT, targeted by miR-149-5p, promotes IL-6-induced inflammatory responses of chondrocytes in osteoarthritis via activating JAK2/STAT3 pathway. *Clin. Exp. Rheumatol.* [Epub ahead of print]. 32141427

[B103] Watterson T. L. Hamilton B. Martin R. Coulombe R. A. (2009). Urban particulate matter causes ER stress and the unfolded protein response in human lung cells. *Toxicol. Sci.* 112 111–122. 10.1093/toxsci/kfp186 19675143

[B104] Wilhelm C. J. Hashimoto J. G. Roberts M. L. Zhang X. Calla M. Bloom S. H. (2018). Plasminogen activator system homeostasis and its dysregulation by ethanol in astrocyte cultures and the developing brain. *Neuropharmacology* 138 193–209. 10.1016/j.neuropharm.2018.06.004 29885422 PMC6310223

[B105] World Health Organization [WHO] (2020). *Laboratory Testing For Coronavirus Disease 2019 (COVID-19) in Suspected Human Cases: Interim Guidance.* Geneva: World Health Organization.

[B106] Wu W. Jin Y. Carlsten C. (2018). Inflammatory health effects of indoor and outdoor particulate matter. *J. Allergy Clin. Immunol.* 141 833–844. 10.1016/j.jaci.2017.12.981 29519450

[B107] Xu G. Jiao L. Zhao S. Yuan M. Li X. Han Y. (2016). Examining the impacts of land use on air quality from a spatio-temporal perspective in Wuhan, China. *Atmosphere* 7:62 10.3390/atmos7050062

[B108] Xu X. Qimuge A. Wang H. Xing C. Gu Y. Liu S. (2017). IRE1α/XBP1s branch of UPR links HIF1α activation to mediate ANGII-dependent endothelial dysfunction under particulate matter (PM) 2.5 exposure. *Sci. Rep.* 7 1–16. 10.1038/s41598-017-13156-y 29044123 PMC5647447

[B109] Yang J. K. Lin S. S. Ji X. J. Guo L. M. (2010). Binding of SARS coronavirus to its receptor damages islets and causes acute diabetes. *Acta Diabetol.* 47 193–199. 10.1007/s00592-009-0109-4 19333547 PMC7088164

[B110] Yi T. Wang J. Zhu K. Tang Y. Huang S. Shui X. (2018). Aryl hydrocarbon receptor: a new player of pathogenesis and therapy in cardiovascular diseases. *Biomed Res. Int.* 2018:6058784. 10.1155/2018/6058784 29984241 PMC6015699

[B111] You X. Gao L. Liu J. Xu C. Liu C. Li Y. (2012). CRH activation of different signaling pathways results in differential calcium signaling in human pregnant myometrium before and during labor. *J. Clin. Endocrinol. Metab.* 97 e1853–e1861. 10.1210/jc.2011-3383 22869609

[B112] Yu B.-Y. Subudeng G. Du C.-G. Liu Z.-H. Zhao Y.-F. Namei E. (2019). Plasminogen activator, tissue type regulates germinal vesicle breakdown and cumulus expansion of bovine cumulus-oocyte complex in vitro†. *Biol. Reprod.* 100 1473–1481. 10.1093/biolre/ioz049 30939202

[B113] Yuan H. Q. Hao Y. M. Ren Z. Gu H. F. Liu F. T. Yan B. J. (2019). Tissue factor pathway inhibitor in atherosclerosis. *Clin. Chim. Acta* 491 97–102. 10.1016/j.cca.2019.01.024 30695687

[B114] Zhang H. Penninger J. M. Li Y. Zhong N. Slutsky A. S. (2020). Angiotensin-converting enzyme 2 (ACE2) as a SARS-CoV-2 receptor: molecular mechanisms and potential therapeutic target. *Intensive Care Med.* 46 586–590. 10.1007/s00134-020-05985-9 32125455 PMC7079879

[B115] Zhang N. Agbor L. N. Scott J. A. Zalobowski T. Elased K. M. Trujillo A. (2010). An activated renin-angiotensin system maintains normal blood pressure in aryl hydrocarbon receptor heterozygous mice but not in null mice. *Biochem. Pharmacol.* 80 197–204. 10.1016/j.bcp.2010.03.023 20359465 PMC2867364

[B116] Zheng L. Huang J. Su Y. Wang F. Kong H. Xin H. (2020). Overexpression of tissue factor pathway inhibitor 2 attenuates trophoblast proliferation and invasion in preeclampsia. *Hum. Cell* 33 512–520. 10.1007/s13577-020-00322-0 32130677

[B117] Zoubovsky S. P. Hoseus S. Tumukuntala S. Schulkin J. O. Williams M. T. Vorhees C. V. (2020). Chronic psychosocial stress during pregnancy affects maternal behavior and neuroendocrine function and modulates hypothalamic CRH and nuclear steroid receptor expression. *Transl. Psychiatry* 10:6. 10.1038/s41398-020-0704-2 32066677 PMC7026416

