# Supplementary material for: The Secretive Liaison of Particulate Matter and SARS-CoV-2. A Hypothesis and Theory Investigation
Source: Front Genet. 2020 Nov 9;11:579964. doi: 10.3389/fgene.2020.579964 (PMC7680895; doi:10.3389/fgene.2020.579964)

Supplementary Figures and Tables


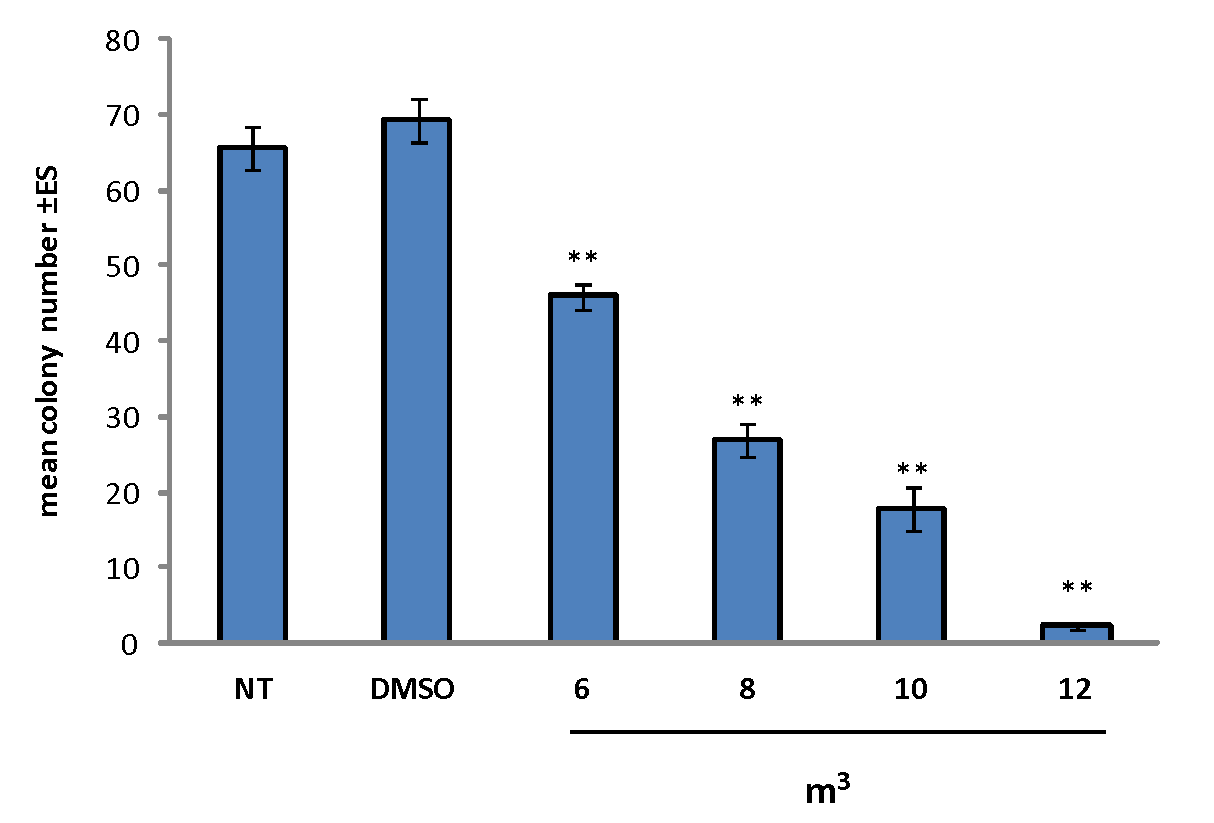


**Fig. S1**. T47D Cytotoxicity assay.

The cytotoxicity assay was performed by exposing the cells to CTW winter samples, representing the worst exposure scenario.

Cells were seeded at 200 cells/plate and exposed to CTW sample. The results are expressed as the mean number of cell colonies growing after 48 hr-treatment (bars = standard error). ** p<0.01, Student *t* test vs DMSO-treated cells

All the assayed doses ( 6, 8, 10, 12 m^3^) , were able to induce significant dose-dependent cytotoxicity. The exposure of T47D cells to the top concentration (12 m^3^) led to the almost total (97%) cell death.


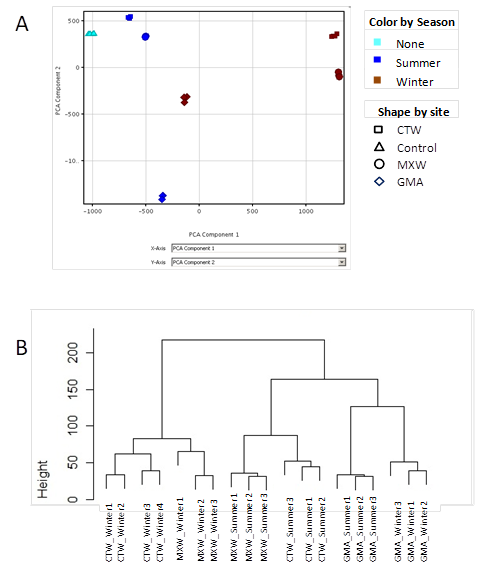


**Fig. S2.** PCA analysis and hierarchical clustering approach to evaluate differences in the transcriptional effects induced by different PM_2.5_ samples. The complete dataset included 23 samples belonging to 7 groups. A = PCA analysis based on 11,483 differentially expressed genes (One-Way ANOVA analysis) in T47D cells. B = Hierarchical Cluster Analysis (Distance: Euclidean; Linkage rule: Ward’s) of the transcriptional profile relative to the entire T47D microarray probe set filtered for low intensity value (log2 intensity value > 3; 29,589 genes).


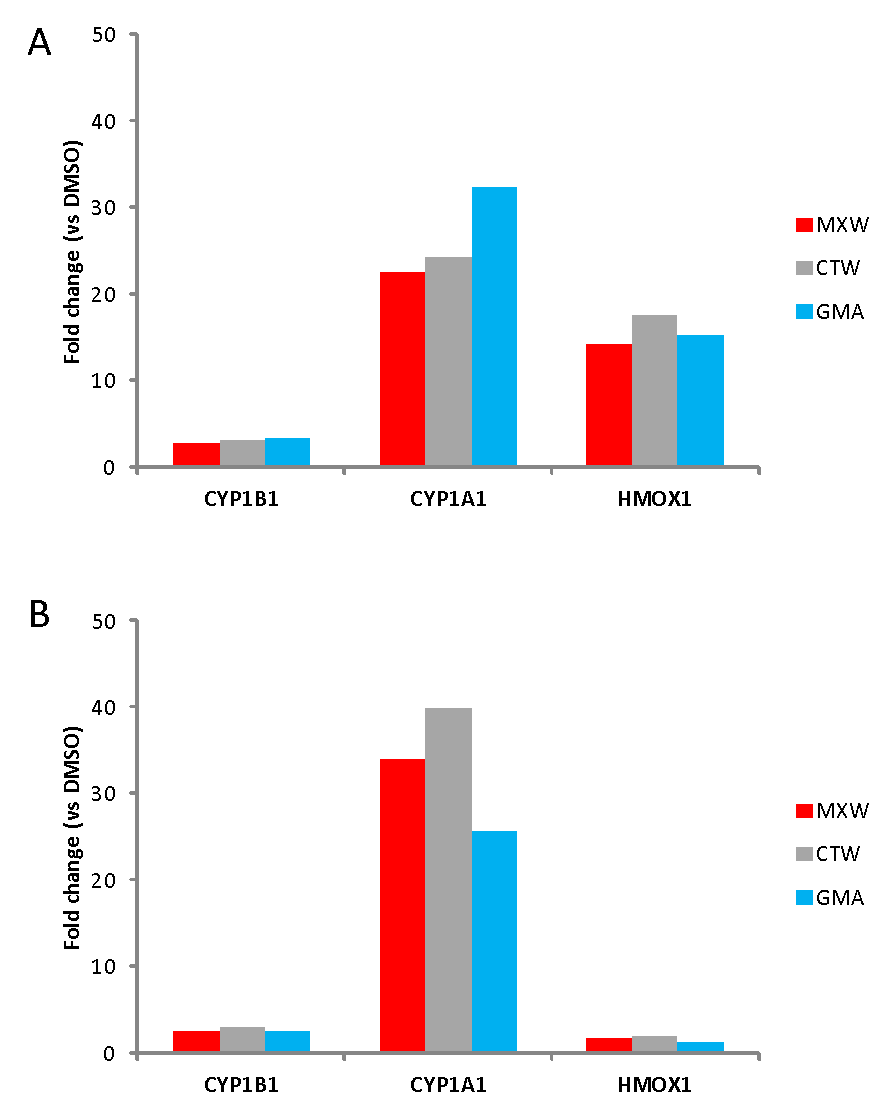


**Fig. S3.** Gene expression intensity profile of CYP1A1, CYP1B1 and HMOX1 compared to the solvent control (0.5% DMSO). (A) winter; (B) summer


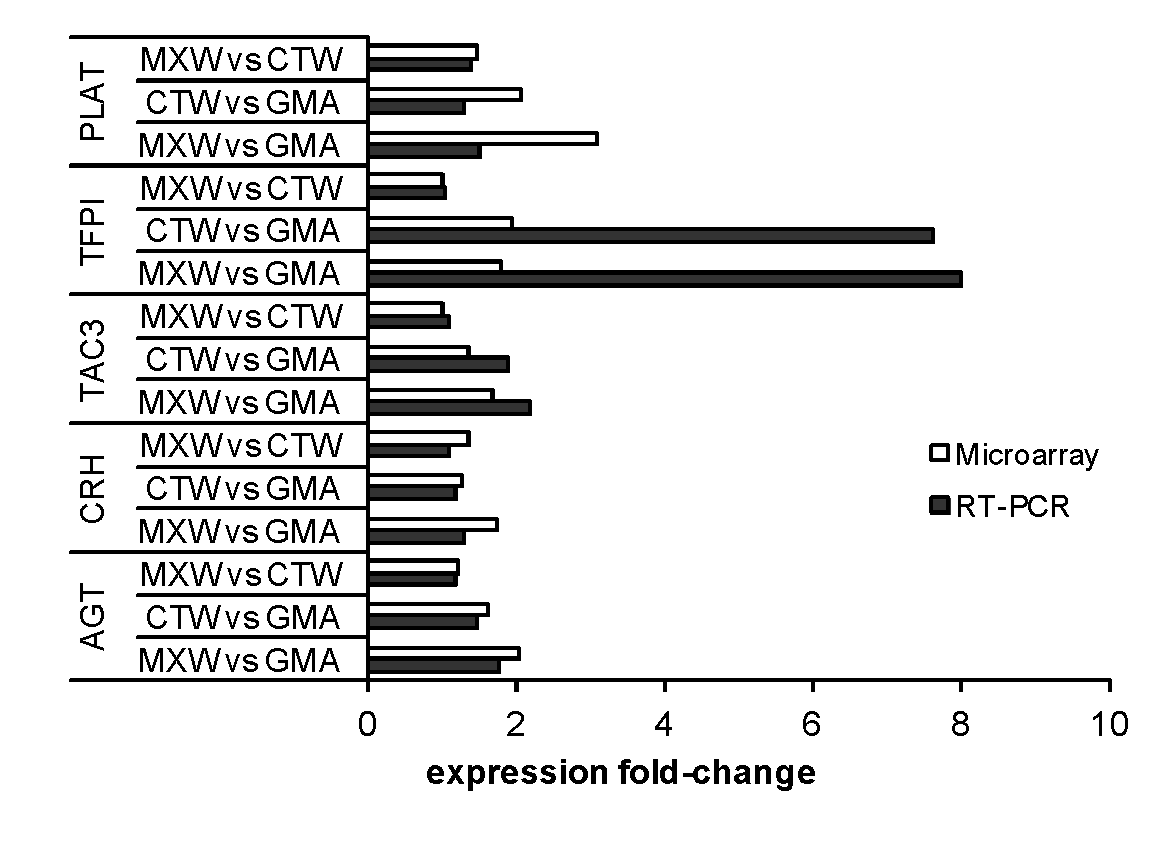


**Fig. S4.** Correlation between microarray data and qRT-PCR data. The qRT-PCR fold changes were calculated by using 2^−ΔΔCt^ method comparing MXW vs CTW, CTW vs GMA and MXW vs GMA.

**Table S1.** Description of the PM samples

| **Name** | **Description** | **Sampling**  **Period** | ${PM}_{2.5}$  **(µg/**$m^{3}$**)** | ${PM}_{2.5}$  **(µg/plate)** | ${PM}_{1}$  **(µg/**$m^{3}$**)** | ${PM}_{1}$  **(µg/plate)** |
| --- | --- | --- | --- | --- | --- | --- |
| **GMA** | Site located in the southern area of Bologna (public park): urban background | Summer | 18.74 | 149.92 | n. d. | n. d. |
|  |  | Winter | 29. 56 | 236.48 | n. d. | n. d. |
| **MXW** | Site located in a surrounding area of Bologna and impacted by the waste-to-energy plant | Summer | 23.33 | 186.64 | n. d. | n. d. |
|  |  | Winter | 35.42 | 283.36 | n. d. | n. d. |
| **CTW** | Site located in a surrounding of Bologna and impacted by all of the same sources as MXW, except the waste-to-energy plant | Summer | 21.11 | 168,88 | n. d. | n. d. |
|  |  | Winter | 34.47 | 275.76 | n. d. | n. d. |

**Table S2.** Content of PM_2.5_ in Winter samples.-


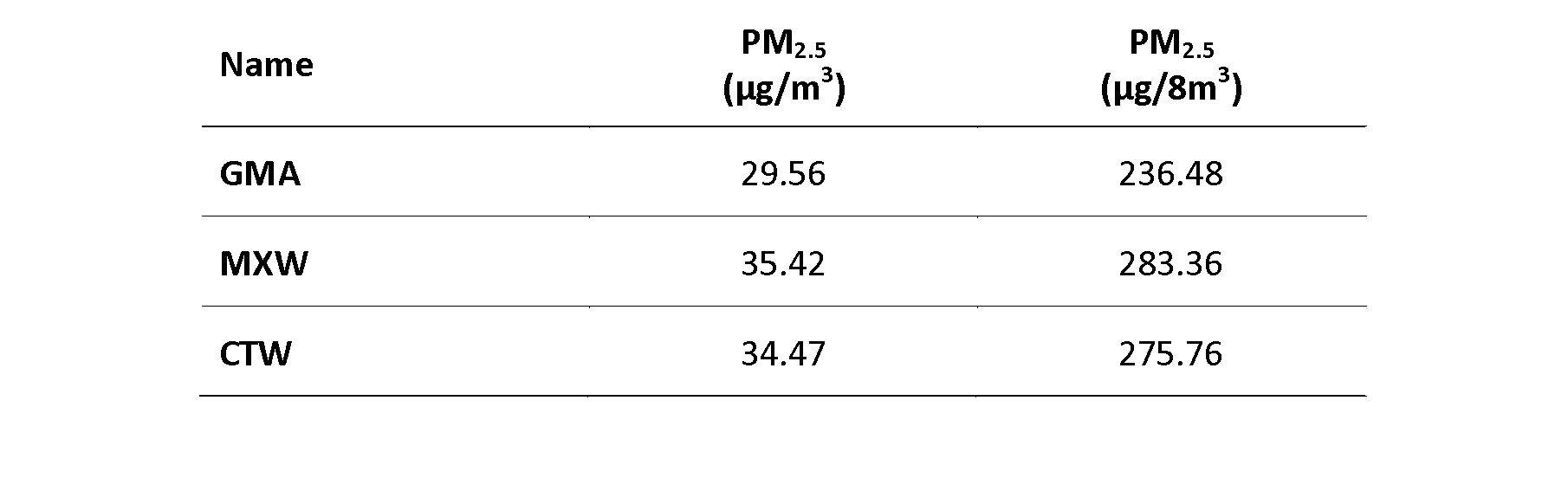


**Table S3.** Number of differentially expressed genes (DEGs) in T47D cells exposed for 4 hrs to PM_2.5_ samples collected in winter (t-test, FDR<0.01, absolute fold-change value greater than 1.2).


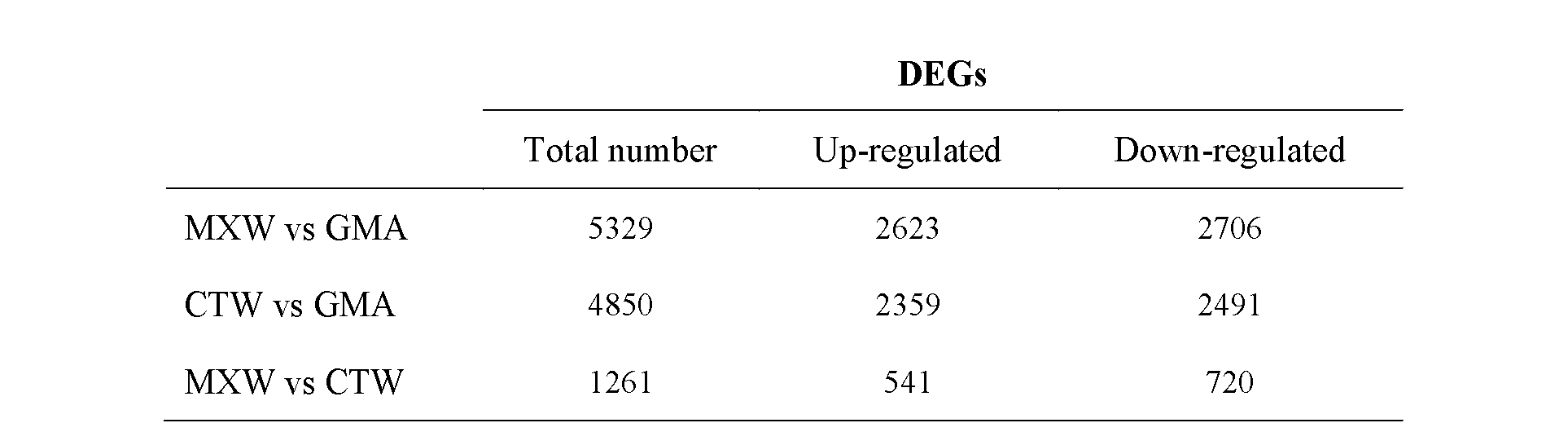


MTX = maximum fall-out site; CTX = minimum-fall out site; GMA = urban background site.

**Table S4.** GSEA analysis - number of KEGG Pathways and GO biological processes enriched in T47D cells exposed to PM winter extracts.


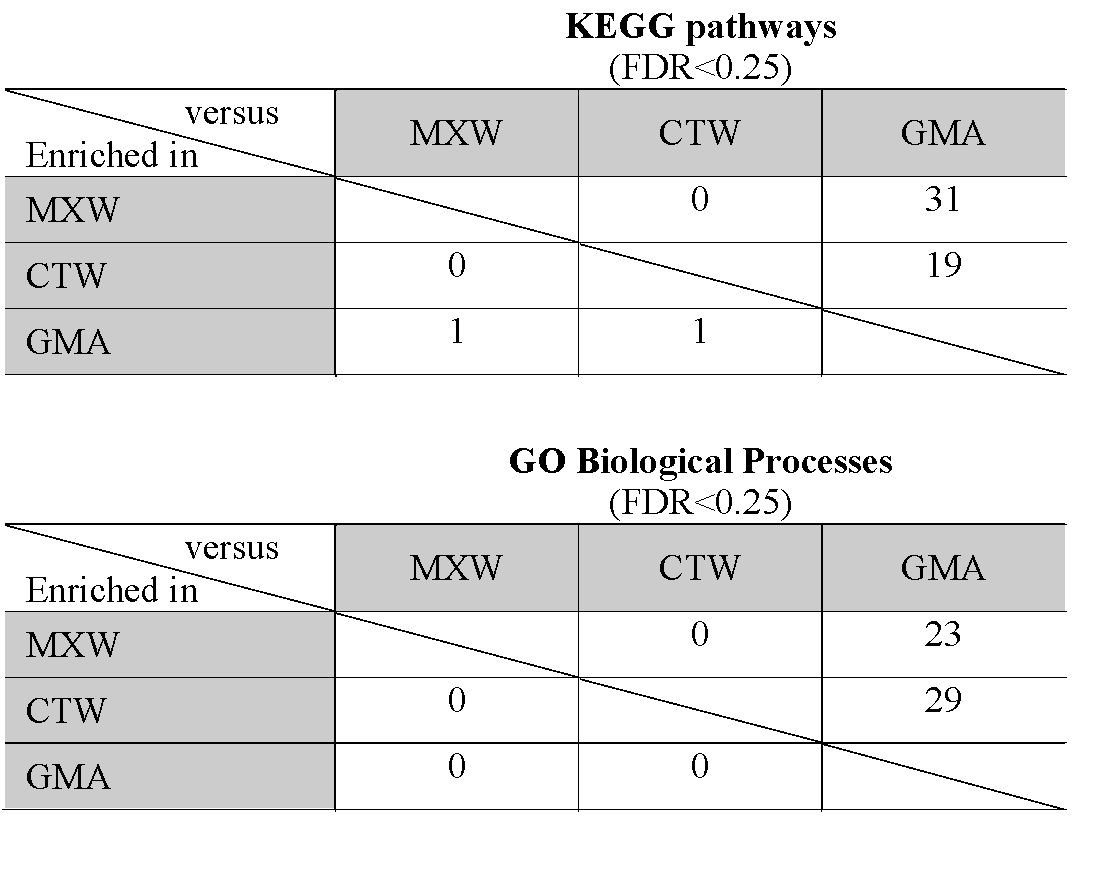

Supplement: Supplementary file 1 [file Data_Sheet_1.docx]
