# Supplementary material for: The Secretive Liaison of Particulate Matter and SARS-CoV-2. A Hypothesis and Theory Investigation
Source: Front Genet. 2020 Nov 9;11:579964. doi: 10.3389/fgene.2020.579964 (PMC7680895; doi:10.3389/fgene.2020.579964)
Supplement: Supplementary file 2 [file Data_Sheet_2.docx]

**Supplementary Materials and Methods**

**Cells**

The T47D cell line was purchased from the American Type Culture Collection (ATCC; Manassas, Virginia). The cells were grown in RPMI-1640 Medium (RPMI) with 0.2 Units/ml bovine insulin and 10% Fetal Bovine Serum (FBS) and routinely maintained in a humidified incubator with an atmosphere of 5% CO_2_ in air at 37 °C. The cell cultures were cryoconserved in culture medium containing 5% dimethyl sulfoxide (DMSO).

**Air samples collection and preparation of treatment solutions**

The PM_2.5_ samples were collected during summer, 2008 (13/06-24/07/2008) and winter, 2009 (14/01-12/03/2009) at several sites located in the surroundings of Bologna (Emilia Romagna, Italy) chosen on the basis of air dispersion models and representative of different levels of environmental pollution. They included: (1) a site typical of the urban background (GMA 25.96 µg/m3), (2) a site affected by the maximum fall-out of the emission of a waste-to-energy plant (MXW 35.42 µg/m3), which was chosen as a punctual source, (3) a site that was considered the minimum fall-out point of the punctual source (CTW 34.47 µg7m3). CTW was located upwind with respect to MXW. Messo anche nel testo (Both MXW and CTW were located in a downwind area, affected by the urban pollution. A PM_2.5_ was collected daily on glass-fiber filters (47 mm diameter) by low-volume air flow samplers (Skypost TCR TECORA). Each filter was weighed daily before and after PM collection in order to obtain the gravimetric data. All the filters collected in one season at each site were pooled to obtain a unique sample that was representative of the season. Each pooled sample was extracted with acetone using a Soxhlet apparatus, to obtain the organic extractable fraction, then reduced to dryness and dissolved in DMSO at a final concentration of 800 m^3^ equivalents/ml.

The treatment solutions were prepared by diluting the stock solutions in the culture media immediately before use. The final concentration of the vehicle was 0.5% DMSO messo anche nel testo

**Preliminary Cytotoxicity Assay**

The cytotoxicity assay was performed by seeding exponentially growing T47D cells at 200 cells/60-mm dish (Falcon, Becton Dickinson, UK), in five replicates for each treatment. Plates were incubated at 37 °C in a 5% CO_2_ humidified atmosphere for 48 h. Cells were then exposed to different concentrations of the environmental samples for 48 h. At the end of this period, the treatment was removed and fresh culture medium was added to the plates. Cells were maintained in culture for 8–10 days, then fixed with methanol, stained with 10% aqueous Giemsa and scored for colony formation. Only colonies containing more than 50 cells were counted. Untreated T47D cells and solvent-treated cells were used as negative controls. The results were expressed as the mean number of colonies/plate ± standard error (SE).

**Cell treatment and total RNA isolation**

T47D cells were seeded at a density 6 x 10^5^ in 60-mm plates and incubated at 37°C in a 5% CO_2_ humidified atmosphere. After 48 h, cells were exposed for 4 h to PM_2.5_ extracts at the concentration of 8 m^3^ equivalents. For PM_2.5_ samples from MXW, CTW and GMA, three biological replicates were performed for each sample using T47D cells from three different cryovials. At the end of the incubation time, total RNA was extracted from each cell sample by using TRIzol Reagent (Life Technologies, Carlsbad, CA, USA) and purified on an RneasyR affinity column (Qiagen, Valencia, CA, USA). RNA quantification and quality were assessed respectively by Nanodrop 1000 (Thermo Scientific, Wilmington, DE, USA) and the Agilent bioanalyzer 2100 using the RNA Nano kit (Agilent Technologies, Santa Clara, CA, USA). Then, at least three technical replicates of microarray experiments were carried out by using pooled mRNA from the biological replicates. Four technical replicates were performed from DMSO or CTW winter samples mRNA.

**Total RNA labeling and hybridization**

Fluorescently-labeled cRNA was generated by using the Low RNA Input Fluorescent Linear Amplification Kit, according to the instructions from the supplier (Agilent Technologies), starting from 1 µg of total RNA derived from cells exposed to MXW, CTW or GMA extracts. Cyanine 3-CTP (Cy-3) (Perkin–Elmer, NEN Life Science, Boston, MA, USA) -labeled cRNAs were purified with QIAquick spin columns (Qiagen) and then applied to the oligonucleotide slides (Whole Human Genome Microarray kit, 4_44K), according to the Agilent 60-mer oligomicroarray processing protocol G4140-90040_One-Color_GE_ version 5.7 (Agilent Technologies).

Slides were scanned in the Cy-3 channel with an Agilent High- Resolution C Scanner (Agilent, G2565AA). Scanned images were analyzed by the Agilent Feature Extraction software version 9.1 to derive the raw intensity data used in the next steps of analysis.

Any other details about the microarray experimental design and protocols, as well as the complete raw data-set are available in the EBI microarray data public repository Arrayexpress <http://www.ebi.ac.uk/arrayexpress/>), accession number E-MEXP-3686

**Statistical Analysis of microarray data**

Raw data were filtered for intensity and quality signal by using GeneSpring GX (Agilent Technologies). All samples were then firstly analyzed with common statistical approach. One-Way ANOVA Analysis (GMA, MXW, CTW, P<0.01 Bonferroni; Main site, P<0.01 Benjamini-Hochberg) was used to select differentially expressed genes among the treatments and the controls. Then, the selected lists were analyzed by Principal Component Analysis (PCA). We flanked the PCA analysis with a Hierarchical Clustering approach using the expression profile of the entire T47D microarray probe set filtered for low intensity values only, without applying any previous statistical analysis.

The analysis of the DEG lists confirmed the modulation of well-known markers of environmental exposures, such as CYP1A1, CYP1B1 and HMOX1, were .

Furthermore, a t-test analysis was carried out to underline the gene modulation induced by each treatment in comparison with control.

For winter organic PM_2.5_ samples MXW vs GMA, CTW vs GMA , MXW vs CTW t tests were performed. “

**Tools of biological interpretation**

The biological analysis of the gene lists derived from the comparison between CTW, MXW and GMA was performed by using Pathway Express (PE, Intelligent Systems and Bioinformatics Laboratory) and by applying a Gene Set Enrichment Analysis (GSEA).

PE is an impact analysis approach which evaluates important parameters such as the expression fold changes of the DEGs as well as the topology of the pathway and the type and strength of the interactions between genes on that pathway. The PE tool provides for each pathway a classical p-value and a p-value derived from the impact analysis (gamma p-value) (Draghici et al, 2007 PMID 17785539).

The Gene Set Enrichment Analysis (GSEA) was carried out, starting from the whole genome dataset without the logarithmic transformation of intensity values and any previous statistical analysis. GSEA is a computational method that determines whether an “a priori” defined set of genes shows statistically significant, concordant differences between two biological states (e.g. phenotypes). GSEA analysis was performed taking advantage of the GSEA Desktop Application downloaded from the Broad Institute site (http://www.broadinstitute.org/gsea/index.jsp) together with the gene set database MSigDB version 3.0. The analysis was restricted to Gene sets belonging to Go Biological Process (http://www.geneontology.org/) with a size comprised from 15 to 500 genes. For each comparison we used the Signal2Noise metric for ranking genes in a descendant order and we applied the “gene based permutation test” to estimate the significance level of the calculated enrichment score by performing 500 permutation tests. By applying the leading edge analysis, it is possible to arrange the list of gene sets that result significantly enriched, allowing the identification of the main biological processes derived from the GSEA analysis. The leading edge subset of a gene set contains the genes that mainly contribute to the set's enrichment score (ES), which reflects the degree of over-representation of a gene set at the top or bottom of a list of genes ranked by their differential expression.

**Quantitative Real-time PCR**

An amount of 750 ng of total RNA was reverse-transcribed at 50°C for 30 min in a 30μl reaction mixture containing SuperScriptTM III Reverse Transcriptase, RNaseOUT Recombinant Ribonuclease Inhibitor, oligo(dT) (2.5 mM), random hexamers (2.5 ng/μl), MgCl2 (10 μM), and NTPs (SuperScriptTM III First-Strand Synthesis Super-Mix for qRT-PCR, Invitrogen). Then 2.5 ng of cDNA were amplified with the appropriate primer pair (200 nM) by using SYBR GreenER qPCR SuperMix for iCycler (Invitrogen) in the same cycling conditions (50°C for 2 min, 95°C for 8 min, 45 cycles at 95°C, 15 s and at 60°C, 60 s). The primers for glyceraldehyde-3-phosphate dehydrogenase (GAPDH), Corticotrophin releasing hormone (CRH), Tissue factor pathway inhibitor (TFPI), Plasminogen activator, tissue (PLAT), Angiotensinogen (AGT), Tachykinin3 (TAC3) were designed with Primer 3 software (http://bioinfo.ut.ee/primer3-0.4.0/primer3/) (Table S1).
